# Relative Selectivity of Plant Cardenolides for Na^+^/K^+^-ATPases From the Monarch Butterfly and Non-resistant Insects

**DOI:** 10.3389/fpls.2018.01424

**Published:** 2018-09-28

**Authors:** Georg Petschenka, Colleen S. Fei, Juan J. Araya, Susanne Schröder, Barbara N. Timmermann, Anurag A. Agrawal

**Affiliations:** ^1^Institute for Insect Biotechnology, Justus-Liebig-Universität, Giessen, Germany; ^2^Department of Ecology and Evolutionary Biology, Cornell University, Ithaca, NY, United States; ^3^Centro de Investigaciones en Productos Naturales, Escuela de Química, Instituto de Investigaciones Farmacéuticas, Facultad de Farmacia, Universidad de Costa Rica, San Pedro, Costa Rica; ^4^Institut für Medizinische Biochemie und Molekularbiologie, Universität Rostock, Rostock, Germany; ^5^Department of Medicinal Chemistry, School of Pharmacy, University of Kansas, Lawrence, KS, United States

**Keywords:** monarch butterfly, Na^+^/K^+^-ATPase, cardenolide, cardiac glycoside, phytochemical diversity, structure–activity relationship, toxin–receptor interaction, resistance

## Abstract

A major prediction of coevolutionary theory is that plants may target particular herbivores with secondary compounds that are selectively defensive. The highly specialized monarch butterfly (*Danaus plexippus*) copes well with cardiac glycosides (inhibitors of animal Na^+^/K^+^-ATPases) from its milkweed host plants, but selective inhibition of its Na^+^/K^+^-ATPase by different compounds has not been previously tested. We applied 17 cardiac glycosides to the *D. plexippus*-Na^+^/K^+^-ATPase and to the more susceptible Na^+^/K^+^-ATPases of two non-adapted insects (*Euploea core* and *Schistocerca gregaria*). Structural features (e.g., sugar residues) predicted *in vitro* inhibitory activity and comparison of insect Na^+^/K^+^-ATPases revealed that the monarch has evolved a highly resistant enzyme overall. Nonetheless, we found evidence for relative selectivity of individual cardiac glycosides reaching from 4- to 94-fold differences of inhibition between non-adapted Na^+^/K^+^-ATPase and *D. plexippus*-Na^+^/K^+^-ATPase. This toxin receptor specificity suggests a mechanism how plants could target herbivores selectively and thus provides a strong basis for pairwise coevolutionary interactions between plants and herbivorous insects.

## Introduction

It is widely recognized that coevolution between plants and herbivores occurs in a community context (Agrawal, 2005; Johnson and Stinchcombe, 2007; Poelman and Kessler, 2016), but the mechanisms and consequences of such complex interactions are unclear. Most plants must defend against multiple herbivores, often from different feeding guilds (Maddox and Root, 1990; Ali and Agrawal, 2014), which poses a challenge for the plant. In some cases, a single defense trait may be effective against a multitude of herbivores. Alternatively, different defenses may defend against alternative herbivores (Linhart and Thompson, 1999; Züst et al., 2012). However, even for relatively well-characterized types of defenses such as certain classes of plant toxins, it is largely unclear whether distinct chemical forms have selective biological activities against different herbivores (Kim and Jander, 2007).

The extend of phytochemical diversity, even within a single compound class and in an individual plant, has long bewildered chemical ecologists (Berenbaum et al., 1991; Jones and Firn, 1991; Berenbaum and Zangerl, 1996; Richards et al., 2015). One potential explanation for this diversity is compound selectivity, that is, individual plant compounds are targeted at distinct herbivores. Selectivity in plant defense has mainly been demonstrated ecologically for induced plant defenses (Agrawal, 2000), and is often driven by trade-offs between signaling cascades and insect feeding guilds (Ali and Agrawal, 2012; Erb et al., 2012; Castillo et al., 2014). Across populations, coevolutionary studies have found some evidence that different herbivores can select for specific defensive traits (Mithen et al., 1995; Züst et al., 2012; Castillo et al., 2014) and it was demonstrated that selective interactions between plant defensive compounds and herbivores exist, i.e., that the same substance can have different effects on different herbivores (Ayres et al., 1997; Linhart and Thompson, 1999). Nonetheless, the underlying mechanisms of such interactions have never been revealed and previous work on natural plant–herbivore interactions has not identified selective toxin–target site interactions. As a first step to address the potential for coevolution between species at the interface of toxins and receptors, one must demonstrate that individual toxins act selectively on targets from different species, i.e., a specific plant toxin is affecting one herbivore’s physiological target relatively stronger or weaker compared to that of another herbivore species.

In this study, we used the specific interaction of cardenolide toxins from plants and insect Na^+^/K^+^-ATPases as a model to test for structure–activity relationships as well as selective interactions as a potential mechanism involved in insect plant coevolution. Due to the ease of interpreting the directly quantified inhibition of Na^+^/K^+^-ATPases *in vitro*, the use of structurally diverse plant toxins which act on the same insect target site represents an unprecedented opportunity to test a set of mechanistic hypotheses. Cardenolides are classic plant toxins in the context of multi-trophic interactions and form a structurally diverse group of compounds which are found in at least 12 plant families (Agrawal et al., 2012). They consist of a steroid core linked to a five-membered lactone ring at C17 and exist either as glycosides or as free genins (Malcolm, 1991). Cardenolides, together with the bufadienolides which carry a six-membered lactone ring, have been termed cardiac glycosides, a name based on their pronounced action on the human heart. In their glycosidic form, one or more sugar molecules bind to position C3. Cardiac glycosides are specific inhibitors of the ubiquitous animal enzyme Na^+^/K^+^-ATPase, which maintains the cellular membrane potential (Yatime et al., 2011).

Cardenolide-producing plants are characterized by the production of diverse cardenolide structures, with foxglove known to produce >100 distinct forms (Luckner and Wichtl, 2000) and many milkweeds producing >20 compounds within individuals of a single species (Agrawal et al., 2012). Comparative studies of structure–activity relationships between cardenolides and the Na^+^/K^+^-ATPase have revealed substantial variation, but have been carried out only in the biomedical context of the vertebrate Na^+^/K^+^-ATPases (Schönfeld et al., 1985; Farr et al., 2002; Paula et al., 2005). Although cardenolides have attracted attention as plant defense compounds against herbivorous insects (Agrawal and Fishbein, 2008; Rasmann et al., 2009), the effect of structural variation on insect Na^+^/K^+^-ATPases has not been previously investigated.

Despite the potent toxicity of cardenolides, many insects have colonized cardenolide-producing plants (Agrawal, 2017) and have evolved mechanisms to protect their Na^+^/K^+^-ATPase from these dietary toxins. Caterpillars of the monarch butterfly (*Danaus plexippus*), for example, are specialized to feed on milkweeds (*Asclepias* spp., Apocynaceae) and sequester plant cardenolides into their hemocoel to store them as a defense against predators (Brower et al., 1967; Reichstein et al., 1968). The monarch butterfly Na^+^/K^+^-ATPase is highly resistant to the standard cardenolide ouabain (Vaughan and Jungreis, 1977), and this resistance is conferred by two amino acid substitutions (L111V and N122H, Holzinger et al., 1992; Aardema et al., 2012). Such target site insensitivity has evolved convergently in at least five insect orders that use cardiac glycoside-producing plants as hosts (Al-Robai et al., 1990; Dobler et al., 2012, 2015; Zhen et al., 2012; Petschenka et al., 2017). Nonetheless, basal milkweed butterfly species in the Danaini, which are adapted to feed on cardenolide-containing plants, maintain a sensitive Na^+^/K^+^-ATPase and have evolved alternative mechanisms to cope with cardenolides (Petschenka et al., 2013a; Petschenka and Agrawal, 2015).

Although monarchs are exposed to a great variety of cardenolides naturally, all previous research on the monarch butterfly Na^+^/K^+^-ATPase is based on the non-milkweed cardenolide ouabain, and no other cardenolide has previously been applied to an insect Na^+^/K^+^-ATPase (Holzinger et al., 1992; Vaughan and Jungreis, 1977; Petschenka et al., 2012). To test the prediction that structurally diverse compounds exert differential pharmacological activities and also may target different herbivore species selectively, here we report on studies where we applied 16 different cardenolides and one bufadienolide to the Na^+^/K^+^-ATPase of the monarch butterfly, a closely related basal milkweed butterfly *Euploea core*, as well as to the Na^+^/K^+^-ATPase of an insect not adapted to dietary cardiac glycosides (the desert locust, *Schistocerca gregaria*). *S. gregaria* Na^+^/K^+^-ATPase likely represents ancestral insect Na^+^/K^+^-ATPase, *E. core*, also maintains a highly sensitive Na^+^/K^+^-ATPase (Petschenka and Agrawal, 2015), and as discussed above, monarchs possess a highly resistant form.

In addition to 11 commercially available cardenolides from non-milkweed sources (which have *cis*–*trans*–*cis* configuration of A/B, B/C, and C/D rings), we also used five *Asclepias* cardenolides (*trans*–*trans*–*cis* configuration) including calactin and calotropin, which are preferentially sequestered by monarchs (Cheung et al., 1988; Groeneveld et al., 1990). We further tested Na^+^/K^+^-ATPase inhibition by eight pregnane glycosides, related steroids occurring alongside cardenolides in *Asclepias* (Araya et al., 2012a) and also *Digitalis* (Teuscher and Lindequist, 2010), but with unknown ecological function. Specifically, we tested if: (1) structural variation of cardenolides causes differences in inhibition of insect Na^+^/K^+^-ATPases, (2) the sugar moiety of cardenolides influences their pharmacodynamic properties, (3) 5β-cardenolides act differently from 5α-cardenolides, the latter of which are typical for *Asclepias* and related genera, (4) individual cardenolides interact selectively with different insect Na^+^/K^+^-ATPases, and (5) if pregnane glycosides have any inhibitory effect on insect Na^+^/K^+^-ATPase.

## Materials and Methods

### Cardenolides and Bufadienolides

**FIGURE 1:**
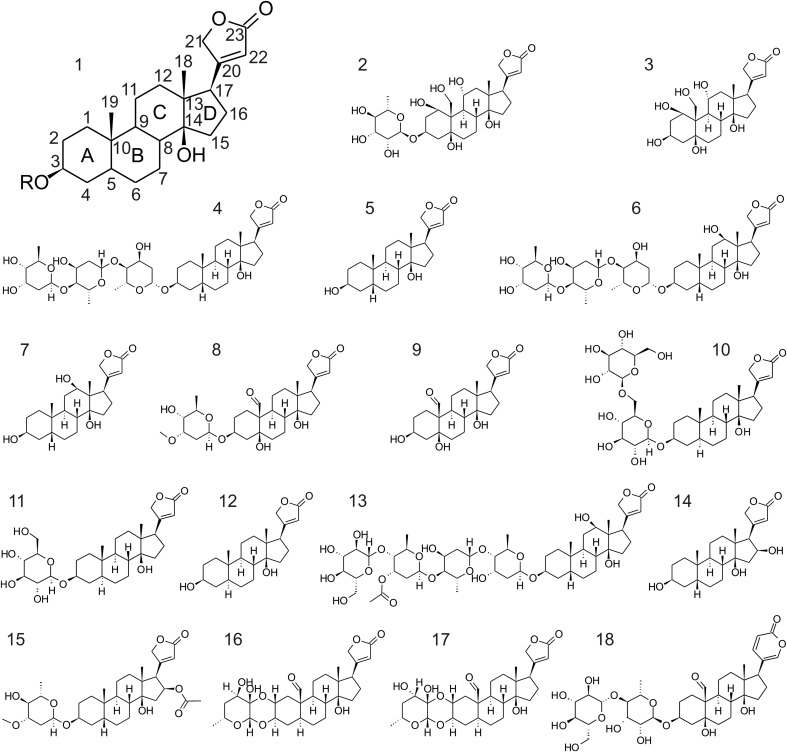
Structural formulas of the cardiac glycosides used in this study. 1, structure of the genin of cardiac glycosides of the cardenolide type (bufadienolides have a six-membered lactone ring as present in 18); 2, ouabain; 3, ouabagenin; 4, digitoxin; 5, digitoxigenin; 6, digoxin; 7, digoxigenin; 8, cymarin; 9, strophanthidin; 10, uzarin; 11, desglucouzarin; 12, uzarigenin; 13, lanatoside C; 14, gitoxigenin; 15, oleandrin; 16, calactin; 17, calotropin; 18, hellebrin.

Most cardiac glycosides used in this study (**Figure 1**) were purchased commercially. Digitoxigenin, digitoxin, digoxigenin, digoxin, gitoxigenin, lanatoside C, ouabagenin, ouabain, and strophanthidin were from Sigma-Aldrich (Taufkirchen, Germany), oleandrin and cymarin were from Phytolab (Vestenbergsgreuth, Germany), uzarigenin from Chem Faces (Wuhan, China), hellebrin from Enzo Life Sciences (Lörrach, Germany), and adynerin from Chromadex (LGC Standards, Wesel, Germany). Uzarin, desglucouzarin as well as the eight pregnane glycosides (verticilloside A-H) were purified from *Asclepias syriaca* and *A. verticillata* previously (Araya et al., 2012a,b).

Calactin and calotropin were purified from caterpillars of *D. plexippus* raised on *A. curassavica* in the course of this study. For this purpose, 15 caterpillars (fifth instar) were frozen in liquid N_2_, freeze-dried, and subsequently dissected to remove gut contents. Dissected tissues were pooled and dried at 50°C overnight in a drying oven. Roughly 1 g of material was extracted with methanol (3 ml × 50 ml) and combined extracts were filtered. After addition of 50 ml deionized water, the solution was extracted with petrol ether (8 ml × 50 ml). Due to poor phase separation, we added additional 50 ml of water and centrifuged. The next day, the recovered methanol–water layer was concentrated in a rotary evaporator until evaporation was very low at 200 mbar (water bath at 60°C). The remaining aqueous phase was extracted five times with ca. 30 ml chloroform, dried with Na_2_SO_4_, filtered, and then evaporated to dryness. The sample was dissolved in methanol and subjected to semi-preparative HPLC. Seventy-five microliters of the extract were injected into an Agilent 1100 series HPLC and compounds were separated on a Nucleodur C18 reversed phase column (5 μm, 250 mm × 10 mm, Macherey-Nagel, Düren, Germany). Cardenolides were eluted on a constant flow of 3 ml/min with an acetonitrile–H_2_O gradient as follows: 0–2 min 16% acetonitrile, 10 min 30% acetonitrile, 25 min 50% acetonitrile, 30 min 50% acetonitrile, and 10 min reconditioning at 1% acetonitrile. UV absorbance spectra were recorded from 200 to 400 nm by a diode array detector. Peaks with symmetrical absorption maxima between 218 and 220 nm at 22.4 and 24.3 min retention time were collected individually. Acetonitrile–water mixtures were dried down to provide crystalline cardenolides. The structural identity of calactin and calotropin was validated by NMR spectroscopy.

### *In vitro* Inhibition Assay of Insect Na^+^/K^+^-ATPase

*Danaus plexippus* was caught in the field or purchased; *E. core* and *S. gregaria* were obtained commercially, with additional *E. core* from a laboratory colony. Na^+^/K^+^-ATPase extractions from butterfly brains or locust recta were prepared as described previously (Petschenka et al., 2013a). Briefly, brains dissected from butterflies stored at -80°C or recta from locusts were homogenized (individually or pooled) with an all glass grinder (Wheaton) in deionized water, frozen at -80°C, freeze-dried, and stored frozen until use. For *in vitro* assays, lyophilisates were dissolved in water, sonicated in an ultrasonic bath, and centrifuged to remove undissolved matter. All cardiac glycosides were tested at least with three biological replicates of Na^+^/K^+^-ATPase (i.e., Na^+^/K^+^-ATPase from genetically different specimens or pools of specimens). For pregnane glycosides, only one biological replicate was collected due to the general lack of pronounced effects.

For estimating the effects of ouabain, ouabagenin, digitoxin, digitoxigenin, digoxin, digoxigenin, cymarin, strophanthidin, lanatoside C, gitoxigenin, oleandrin, and hellebrin on Na^+^/K^+^-ATPase of *D. plexippus* and *S. gregaria*, all Na^+^/K^+^-ATPase inhibition assays were carried out in duplicate (i.e., three biological and six technical replicates per cardiac glycoside tested). Means of duplicate determinations (i.e., technical replicates) were used as data points. In consequence, each data point of a dose–response curve is the mean of at least three biological replicates based on two technical replicates, each. In a few cases (digitoxigenin, lanatoside C, oleandrin, and hellebrin on *D. plexippus*-Na^+^/K^+^-ATPase) more than six technical replicates were collected. Here, technical replicates collected on the same 96-well plate were averaged and used as data points. According to our previous work on cardiac glycoside resistance of Na^+^/K^+^-ATPase from different insect orders, we do not expect intraspecific variation. Therefore, all variation observed within a species should represent technical noise and discrimination between biological and technical replication will not influence the results. Technical replication was omitted for determination of the effects of uzarin, desglucouzarin, uzarigenin, digitoxigenin, calactin, and calotropin on *D. plexippus* and *E. core*-Na^+^/K^+^-ATPase.

**FIGURE 2:**
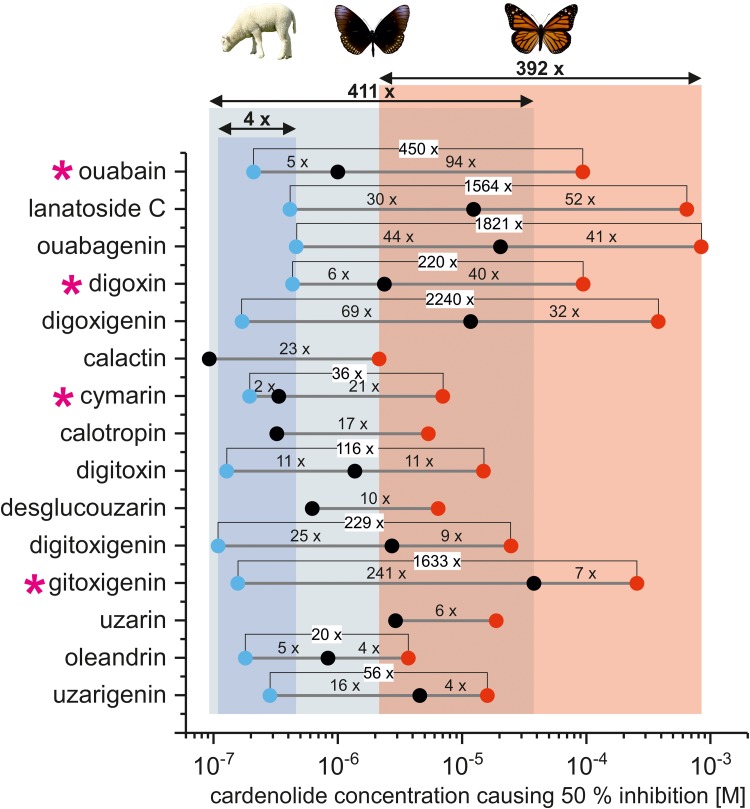
IC_50_ values of individual cardiac glycosides applied to lamb [blue dots, based on (Paula et al., 2005)], non-adapted (black dots), and adapted insect Na^+^/K^+^-ATPase (red dots). Numbers above gray lines indicate the fold difference between the IC_50_ values of individual cardiac glycosides for all three forms of Na^+^/K^+^-ATPase. The overall variation of IC_50_ values for individual cardenolides across each form of Na^+^/K^+^-ATPase is indicated by columns (blue = lamb, gray = *E. core*/*S. gregaria*, and red = *D. plexippus*) and double arrows (numbers above arrows indicate fold differences). Pink asterisks highlight cardenolides which show especially high discrimination between lamb, non-adapted, and adapted insect Na^+^/K^+^-ATPase. Note that for calactin, calotropin, desglucouzarin, and uzarin no data for lamb Na^+^/K^+^-ATPase were available.

**FIGURE 3:**
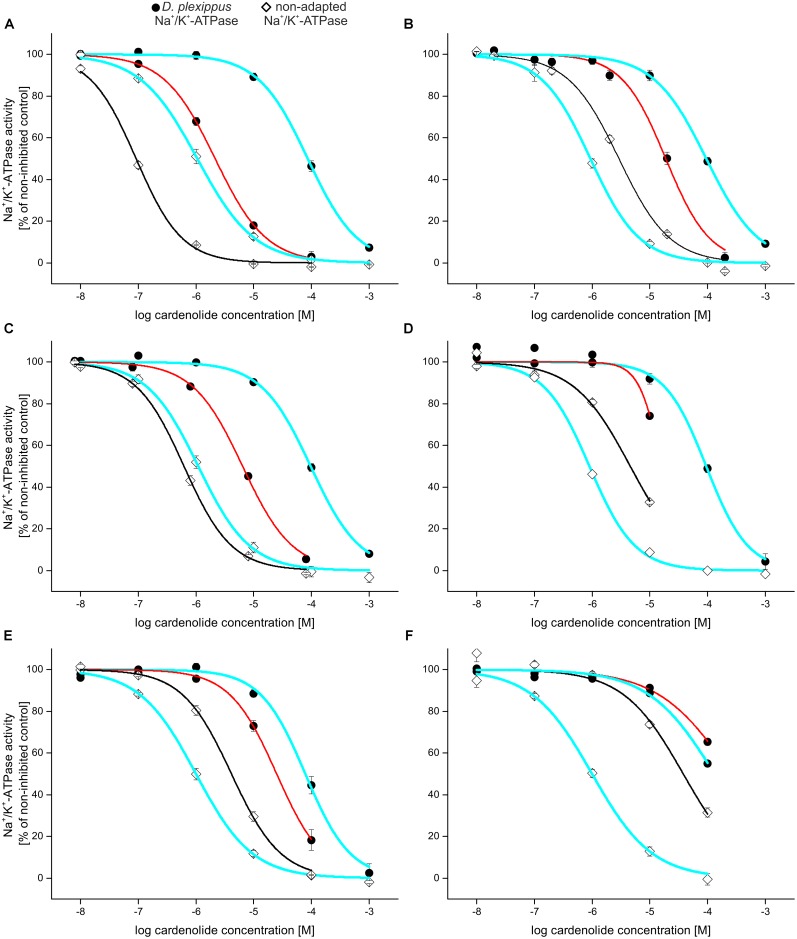
Relative selectivity of inhibition visualized by dose–response curves: inhibition of Na^+^/K^+^-ATPases of *D. plexippus* (circles), *E. core* [diamonds **(A–E)**], and *S. gregaria* [diamonds, **(F)**] by selected cardenolides compared to ouabain (bold blue line; left curve = *S. gregaria*/*E. core*-Na^+^/K^+^-ATPase, black; right curve = *D. plexippus*-Na^+^/K^+^-ATPase, red). **(A)** Calactin, **(B)** uzarin, **(C)** desglucouzarin, **(D)** uzarigenin, **(E)** digitoxigenin, and **(F)** gitoxigenin. Note that adapted and non-adapted Na^+^/K^+^-ATPases are differentially impaired relative to ouabain.

Na^+^/K^+^-ATPase activity was quantified by photometric determination of inorganic phosphate released from enzymatic ATP hydrolysis compared to a non-inhibited control (Petschenka et al., 2013a). The sensitive insect Na^+^/K^+^-ATPases of *S. gregaria* and *E. core* showed no differences based on inhibition by ouabain (*F*_2,7_ = 0.29, *p* = 0.75) and digitoxigenin (*F*_2,6_ = 3.29, *p* = 0.11). As both enzymes also are identical at positions 111 and 122 (S. Dobler, personal communication, April 2017) known to be critical for cardenolide resistance, results from both Na^+^/K^+^-ATPases were combined for some analyses (**Figures 2** and **3**; **Supplementary Figures S3**–**S5**). For **Figure 2** and **Supplementary Figures S3**–**S5**, the IC_50_ values for digitoxigenin and ouabain obtained with *S. gregaria*-Na^+^/K^+^-ATPase were used.

### Preparation of Inhibitors (Cardiac Glycosides and Pregnane Glycosides)

Inhibitors were typically dissolved in 10% dimethyl sulphoxide (DMSO) in water resulting in a final concentration of 2% DMSO in the assay. For the 5α-cardenolide uzarigenin, we used 25% DMSO in water (final concentration in the assay 5%) as we observed at least three to four times lower solubility of uzarigenin compared to its 5-β-isomer digitoxigenin under ambient conditions. For the comparison of uzarigenin and digitoxigenin, the digitoxigenin was also dissolved in 25% DMSO in water. Inhibitor concentrations typically ranged from 10^-4^ to 10^-8^ M with the exception of digitoxin, oleandrin, and uzarigenin whose solubility was too low to achieve 10^-4^ M in our solvent system. Stock solutions of cardiac glycosides, as well as pregnane glycosides, were prepared from crystalline compounds in DMSO. While concentrations of all other compounds were based on mass, calactin, and calotropin stock solutions were adjusted with HPLC using digitoxin as a reference. Diluted inhibitor solutions of uzarin, desglucouzarin, calactin, and calotropin (5 × 10^-4^ and 5 × 10^-5^ M) were additionally surveyed with HPLC (using digitoxin as a reference).

### *In vitro* Inhibition Assay of Porcine Na^+^/K^+^-ATPase

For comparison with insect Na^+^/K^+^-ATPase, we also tested inhibition of the cardenolides ouabain, desglucouzarin, and uzarin on the porcine Na^+^/K^+^-ATPase (Sigma-Aldrich, St. Louis, MO, United States) which was purified from pig brain. Lyophilized Na^+^/K^+^-ATPase was dissolved in water to a concentration of 1 U/ml, stored at -80°C in single-use aliquots and diluted with H_2_O to 0.05 U/ml for use in the *in vitro* assay (final concentration in the assay 0.01 U/ml). The data on inhibition of porcine Na^+^/K^+^-ATPase are presented in **Supplementary Figure S7** and **Supplementary Table S1**.

### Data Evaluation

Non-linear curve fitting of dose–response curves was carried out using OriginPro 2016 (OriginLab Corporation, Northampton, MA, United States) with top and bottom asymptotes set to 100 and 0, respectively. Pairwise statistical comparisons of dose-response curves were carried out using the “compare datasets” option in OriginPro, which is based on an *F*-Test evaluating if the two curves are identical. Correlation analyses using log10-transformed IC_50_ values was also carried out with OriginPro. To illustrate selective effects of individual cardiac glycosides on different forms of Na^+^/K^+^-ATPase (adapted or non-adapted), inhibition was compared to a reference cardenolide (**Supplementary Figures S3**, **S4**). For this purpose, we computed the log10 of the ratio (IC_50_ reference compound)/(IC_50_ test compound). We used ouabain as a reference as it is the most widely used cardenolide in research on Na^+^/K^+^-ATPase (Paula et al., 2005). This procedure results in negative values for compounds inhibiting Na^+^/K^+^-ATPase stronger than ouabain and positive values for compounds inhibiting Na^+^/K^+^-ATPase weaker than ouabain. The results of these comparisons strongly depend on the cardiac glycoside used as a reference. Therefore, in addition to ouabain, we used one of the least toxic cardiac glycosides for both forms of Na^+^/K^+^-ATPase (ouabagenin), an intermediate inhibitor (digitoxigenin), and the most toxic cardiac glycoside (calactin) as reference toxins (**Supplementary Figure S4**). To analyze relative selectivity without a reference, we also plotted the fold differences between the IC_50_ values of individual cardenolides for non-adapted and adapted insect Na^+^/K^+^-ATPase and compared them to mammalian (lamb) Na^+^/K^+^-ATPase (**Figure 2**). IC_50_ values for lamb Na^+^/K^+^-ATPase were inferred from the study of Paula et al. (2005). We excluded seven outliers out of 1,700 absorbance measurements which were clearly due to technical errors. The data reported in this paper are available from the Dryad Digital Repository (Petschenka et al., 2018).

## Results

### Structural Variation Causes Differential Inhibition of Adapted and Non-adapted Na^+^/K^+^-ATPases

Pairwise comparisons of all cardenolide glycosides (**Figure 1**) applied to both the monarch- and one of the non-adapted insect Na^+^/K^+^-ATPases (*S. gregaria* or *E. core*) revealed that the *D. plexippus*-Na^+^/K^+^-ATPase was 4- to 94-fold more resistant (based on IC_50_ values, **Figure 2**, please see **Supplementary Table S1** for a complete list of IC_50_ values), and the level of enhanced resistance depended strongly on the specific compound tested (**Figure 2** and see below).

When effects of the same cardenolides were compared on Na^+^/K^+^-ATPases from a vertebrate species (lamb, Paula et al., 2005), *S. gregaria* or *E. core*, and monarchs, we observed much less variation (fourfold) in the IC_50_ values of the more sensitive mammalian enzyme compared to either of the insect Na^+^/K^+^-ATPases (>100-fold variation, **Figure 2**). Inclusion of additional cardenolides for which no data on lamb Na^+^/K^+^-ATPase were available revealed roughly 400-fold variation in IC_50_ values (**Figure 2**) for both insect Na^+^/K^+^-ATPases.

Overall, the IC_50_ values ranged from 8.42 × 10^-7^ (hellebrin) to 8.41 × 10^-4^ M (ouabagenin) for the *D. plexippus*-Na^+^/K^+^-ATPase and from 9.20 × 10^-8^ M (calactin) to 3.78 × 10^-5^ M (gitoxigenin) for non-adapted insect-Na^+^/K^+^-ATPases (**Supplementary Table S1** and **Figure 2**). Although the bufadienolide hellebrin was the most toxic cardiac glycoside for the *D. plexippus*-Na^+^/K^+^-ATPase overall, the most toxic cardenolide for both Na^+^/K^+^-ATPases tested was calactin (IC_50_ *E. core*-Na^+^/K^+^-ATPase = 9.20 × 10^-8^ M, IC_50_ *D. plexippus*-Na^+^/K^+^-ATPase = 2.15 × 10^-6^ M). The eight pregnanes applied on *E. core*- and *D. plexippus*-Na^+^/K^+^-ATPase (**Supplementary Figure S1**) resulted in only slight inhibition (ca. 90% remaining activity at 10^-4^ M in both species) which is in agreement with an observed lack of activity on porcine Na^+^/K^+^-ATPase (Komarnytsky et al., 2013). Furthermore, there was no difference in the effect of pregnanes between the Na^+^/K^+^-ATPases of *E. core* and *D. plexippus* (**Supplementary Figure S1**). One cardenolide, adynerin, which is known to lack cardiotonic activity in vertebrates (Imai et al., 1965) also failed to inhibit the *S. gregaria*-Na^+^/K^+^-ATPase (**Supplementary Figure S2**).

### Different Cardiac Glycosides Show Relative Selectivity for Different Insect Na^+^/K^+^-ATPases

Comparison of the IC_50_ values of individual cardiac glycosides for lamb, non-adapted, and adapted insect Na^+^/K^+^-ATPases (**Figure 2**) revealed strong relative selectivity exerted by some of the inhibitors tested. Between non-adapted insect and *D. plexippus*-Na^+^/K^+^-ATPase, the polar cardenolide ouabain showed the strongest relative selectivity with a 94-fold difference between the two IC_50_ values. By contrast, IC_50_ values for oleandrin and uzarigenin differed only by fourfold indicating much lower discrimination between adapted and non-adapted insect Na^+^/K^+^-ATPase. Across lamb, non-adapted and adapted insect Na^+^/K^+^-ATPase, cardenolides such as ouabagenin or digitoxin discriminated more or less equally between the three forms of Na^+^/K^+^-ATPase. Ouabain and cymarin, on the other hand, discriminate strongly between non-adapted and *D. plexippus*-Na^+^/K^+^-ATPase but to a much lesser extent between lamb and non-adapted insect Na^+^/K^+^-ATPase. This effect seems not to be related to the overall level of Na^+^/K^+^-ATPase resistance as indicated by the pronounced selectivity of gitoxigenin between lamb and non-adapted insect Na^+^/K^+^-ATPase and the relatively low discrimination of this compound between non-adapted insect and *D. plexippus*-Na^+^/K^+^-ATPase.

To illustrate selective interactions on the level of toxins and receptors, we plotted full dose–response curves for adapted vs. non-adapted Na^+^/K^+^-ATPases and different cardiac glycosides relative to a standard, ouabain (**Figure 3**). Calactin, for example, inhibited *D. plexippus*- as well as *E. core*-Na^+^/K^+^-ATPase more potently than ouabain (**Figure 3A**). By contrast, the diglucoside uzarin (**Figure 3B**) caused a substantially stronger inhibition than ouabain on *D. plexippus* Na^+^/K^+^-ATPase, but far weaker inhibition than ouabain on *E. core* Na^+^/K^+^-ATPase while desglucouzarin (**Figure 3C**) which has the same genin, but linked to only one glucose, had a strongly inhibitory effect on *D. plexippus*-Na^+^/K^+^-ATPase compared to ouabain, and had nearly the same inhibitory effect as ouabain on the *E. core*-Na^+^/K^+^-ATPase. The application of uzarigenin, the genin of this series, and also its 5β isomer digitoxigenin again resulted in countervailing effects on the two insect Na^+^/K^+^-ATPases compared to ouabain (**Figures 3D,E**). In contrast to desglucouzarin, gitoxigenin inhibited the non-adapted locust-Na^+^/K^+^-ATPase to a much lower extent compared to ouabain, whereas the inhibition of *D. plexippus*-Na^+^/K^+^-ATPase was similar to the effect caused by ouabain (**Figure 3F**).

Across the 14 comparisons made in total (**Supplementary Figure S3** and **Supplementary Table S2**), 10 compounds caused a unidirectional effect, i.e., the effect of the compound on the enzyme compared to ouabain was similar when tested on both forms of Na^+^/K^+^-ATPase. The remaining four toxins, however, produced countervailing effects, e.g., stronger than ouabain on the adapted Na^+^/K^+^-ATPase but weaker (or equal) to ouabain on the non-adapted Na^+^/K^+^-ATPase. We emphasize that the observed patterns (i.e., stronger or weaker toxicity compared to the reference) strongly depend on the reference compound used and cannot be viewed in absolute terms. Using ouabagenin, digitoxigenin, or calactin as a reference in place of ouabain shifted the direction of the effects but nonetheless supports visualization of relative selectivity exposed by individual cardiac glycosides on the two different forms of insect Na^+^/K^+^-ATPase (**Supplementary Figure S4**).

To test for the occurrence of such specific interactions more broadly, we carried out correlation analysis between inhibition caused by the same 11 cardiac glycosides on the adapted *D. plexippus*-Na^+^/K^+^-ATPase, the non-adapted Na^+^/K^+^-ATPase of *E. core* and *S. gregaria*, and mammalian Na^+^/K^+^-ATPase from lamb (Paula et al., 2005). Along this gradient of resistance (*D. plexippus* > *E. core*/*S. gregaria* > lamb, **Figure 2**), we observed a positive correlation between the IC_50_ values for cardenolides applied to non-adapted insect- and *D. plexippus*-Na^+^/K^+^-ATPase (**Supplementary Figure S5A**, Pearson’s *r* = 0.82, *n* = 11, *p* = 0.002), but no correlation between the IC_50_ values for cardenolides applied to lamb Na^+^/K^+^-ATPase (Paula et al., 2005) and either of the insect-ATPases (**Supplementary Figures 5B,C**, EC/SG: *r* = 0.28, *n* = 11, *p* = 0.401; DP: *r* = 0.49, *n* = 11, *p* = 0.125). These results indicate that, on a broad scale, the extent of inhibition is not predictable between the vertebrate and insect Na^+^/K^+^-ATPases, but there is greater concordance among insect Na^+^/K^+^-ATPases, even with clear differences in the enzyme.

### Comparison of Structural Attributes and Na^+^/K^+^-ATPase Inhibition

**FIGURE 4:**
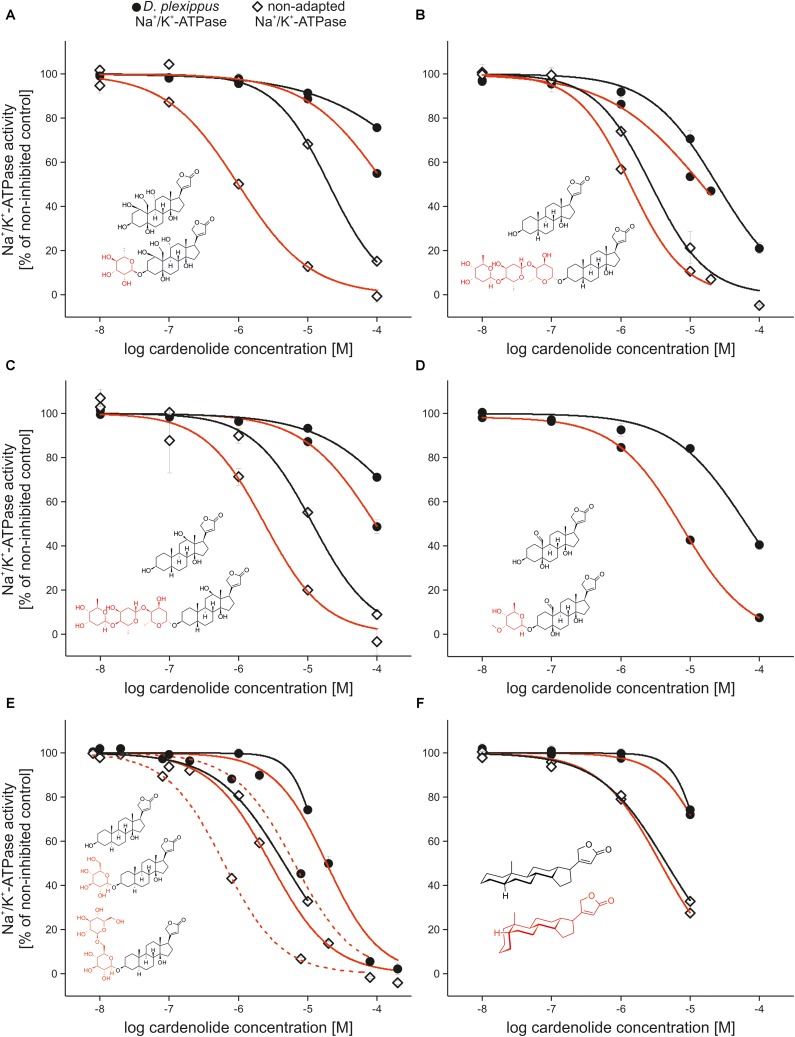
Comparison of the inhibition caused by cardiac glycosides and corresponding genins and the effect of 5α vs. 5β configuration. **(A–E)** Inhibition of Na^+^/K^+^-ATPases of *Danaus plexippus* (circles), *Euploea core* and *Schistocerca gregaria* (diamonds) by cardenolide glycosides (black lines), and their corresponding genins (red lines). Inserted structural formulas show the glycosides and genins used for comparisons. Sugar moieties of glycosides are labeled in red. **(A)** Ouabain vs. ouabagenin, **(B)** digitoxin vs. digitoxigenin, **(C)** digoxin vs. digoxigenin, and **(D)** cymarin vs. strophanthidin. All comparisons are made between *D. plexippus* and *S. gregaria*-Na^+^/K^+^-ATPase. **(E)** Effect of uzarin (solid red line) vs. desglucouzarin (dashed line) vs. uzarigenin (solid black line) on *D. plexippus*- (circles) and *E. core*-Na^+^/K^+^-ATPase (diamonds). **(F)** Inhibition of *D. plexippus* (circles) and *E*. *core*-Na^+^/K^+^-ATPase (diamonds) by digitoxigenin (red line) and its 5α-isomer uzarigenin (black line). The insert shows the steroidal backbone plus lactone of cardiac glycosides with A/B rings in *trans* (black, as realized in uzarigenin) and A/B rings in *cis* configuration (red, as realized in digitoxigenin).

We next compared the inhibition of Na^+^/K^+^-ATPase using five pairs of cardenolide glycosides and their corresponding genins, clearly demonstrating that glycosides are consistently more potent inhibitors of the enzyme (**Figures 4A–E**, see **Supplementary Table S3** for IC_50_ values and statistics). The comparison of the uzarin–desglucouzarin–uzarigenin series of identical compounds with varying numbers of sugar groups revealed that *E. core* and *D. plexippus* Na^+^/K^+^-ATPases were most strongly inhibited by the monoglucoside desglucouzarin (**Figure 4E** and **Supplementary Table S3**). The biglucoside uzarin caused weaker inhibition than desglucouzarin (DP: *F*_2,6_ = 27.9, *p* < 0.001; EC: *F*_2,6_ = 71.371, *p* < 0.001) and was not statistically different from the genin uzarigenin (**Supplementary Table S3**). Thus, the number of sugar groups attached to a cardenolide does not necessarily correspond to the extent of Na^+^/K^+^-ATPase inhibition.

Most *Asclepias* cardenolides have an A/B-*trans*-steroid skeleton while cardenolides from other genera typically have an A/B-*cis* configuration (Seiber et al., 1983; Cheung et al., 1988; Malcolm, 1991). We compared the inhibition of *E. core* and *D. plexippus* Na^+^/K^+^-ATPases by the 5α-cardenolide uzarigenin and its 5β equivalent, digitoxigenin. As for the other cardiac glycosides described above, we found that both, uzarigenin and digitoxigenin, inhibited *E. core* Na^+^/K^+^-ATPase more strongly than *D. plexippus* Na^+^/K^+^-ATPase. However, we observed no difference between uzarigenin and digitoxigenin in terms of their ability to inhibit either of the Na^+^/K^+^-ATPases (**Figure 4F**, DP: *F*_2,4_ = 0.898, *p* = 0.476, EC: *F*_2,4_ = 1.942, *p* = 0.257).

## Discussion

Here, we have shown that structural variation in plant toxins, belonging to the same chemical class, strongly influences the strength of inhibition of the specific target site. Differential activity of structurally diverse defensive metabolites and selective impairment of different herbivores is a major prediction of coevolutionary theory and has been demonstrated on the level of whole organisms (Ayres et al., 1997; Linhart and Thompson, 1999; Müller et al., 2010; Kleine and Müller, 2011). The underlying mechanisms, however, have rarely been revealed, especially on divergent targets of adapted and non-adapted herbivore species. Monarch butterflies have been assumed to be highly resistant to cardenolides by means of an altered Na^+^/K^+^-ATPase, although the effects are dose-dependent and were only previously tested with ouabain, a cardenolide that does not occur in the monarch’s host plants (Vaughan and Jungreis, 1977; Holzinger et al., 1992; Petschenka et al., 2012). Our comparison of 16 cardiac glycosides (including *Asclepias* cardenolides) on *D. plexippus*-Na^+^/K^+^-ATPase is thus a critical step to approach the complex and evolutionarily relevant scenario monarchs and other milkweed herbivores are exposed to naturally.

While the *D. plexippus*-Na^+^/K^+^-ATPase is consistently more resistant than the non-adapted insect Na^+^/K^+^-ATPases of *E. core* and *S. gregaria* (**Figure 2** and **Supplementary Table S1**), 11 out of 16 cardenolides tested acted stronger on its Na^+^/K^+^-ATPase than ouabain. Strikingly, calactin and calotropin, which are preferentially sequestered by monarchs from *Asclepias* spp. (Cheung et al., 1988; Groeneveld et al., 1990; **Supplementary Figure S6**), were the most toxic cardenolides and inhibited *D. plexippus*-Na^+^/K^+^-ATPase up to 60 times more strongly than ouabain (based on IC_50_ values). As *A. curassavica* is a rich source of calactin and calotropin (**Supplementary Figure S6**) and also a very good dietary resource for monarch caterpillars (Petschenka and Agrawal, 2015), it is unlikely that this observed *in vitro* enzymatic toxicity causes a substantial physiological burden. As these compounds can be detected in the hemolymph, the monarch probably has additional mechanisms of resistance (e.g., barrier tissues preventing contact with the *D. plexippus*-Na^+^/K^+^-ATPase, Petschenka et al., 2013b). Monarch caterpillars sequester calactin and calotropin in high amounts and additionally derive these compounds enzymatically from other milkweed cardenolides such as uscharidin (Seiber et al., 1980). Moreover, monarchs only seem to sequester cardenolides within a certain polarity range and compounds such as calactin and calotropin may be easier to store in the body tissues compared to more lipophilic cardenolides (Roeske et al., 1976). Duffey (1977) stated that while non-polar cardenolides are absorbed more easily via the gut relatively more polar cardenolides seem to cause emesis in vertebrates at lower doses. While the moderately non-polar cardenolides calactin and calotropin are easily absorbed across the gut they also have low-dose requirements to cause emesis after reaching the blood stream of a vertebrate (see also Parsons and Summers, 1971). Thus, calactin and calotropin seem to represent the ideal substrate for sequestration, which is supported by observations on the grasshopper *Poekilocerus bufonius* that also selectively sequesters calactin and calotropin (Roeske et al., 1976). It is unknown whether these compounds are highly bitter or easily detected by taste for would-be predators.

The different cardiac glycosides tested produced much higher variation of IC_50_ values when applied to adapted and non-adapted insect Na^+^/K^+^-ATPases (**Figure 2**) compared to the more sensitive lamb-Na^+^/K^+^-ATPase which showed “little fine-specificity of binding” (Farr et al., 2002). While our data do not allow for drawing specific conclusions about monarch–milkweed coevolution, our results demonstrate that the more resistant insect Na^+^/K^+^-ATPases respond variably to structurally distinct cardiac glycosides, opening the potential for coevolution mediated by toxin–receptor interactions. While synergistic effects of different compounds on herbivores were suggested to select for phytochemical diversity (Berenbaum et al., 1991; Dyer et al., 2003), adapted insect target sites could also impose natural selection on the plant to maintain structurally different toxins. This increased diversity of compounds would then raise the probability to possess a toxin which is an especially potent inhibitor for this particular form of the enzyme target. Given the dramatically higher absolute amounts of toxins necessary to inhibit an adapted Na^+^/K^+^-ATPase, using the most potent cardiac glycosides for defense may be especially critical to save costs of production for the plant (Züst et al., 2015).

It was historically shown that cardenolide glycosides are more toxic than corresponding genins in whole organism vertebrate assays (Hoch, 1961). Sugar residues of cardiac glycosides have been shown to stabilize the Na^+^/K^+^-ATPase-inhibitor complex by interactions of hydroxyl groups of the sugar molecule with proton-donating, as well as proton-accepting, groups on the Na^+^/K^+^-ATPase (Yoda, 1973) and sugars can prevent reactivation of the enzyme after cardiac glycoside inhibition (Cornelius et al., 2013). Furthermore, fixation of cardiac glycosides to the heart muscle depends on the sugar moiety (Hoch, 1961). In general, the effect of sugars on any given Na^+^/K^+^-ATPase seem to depend on the number and chemical identity of the sugar molecules (Farr et al., 2002). For the vertebrate Na^+^/K^+^-ATPase, responses were heterogeneous and the removal of sugar is reported to decrease or increase strength of inhibition (O’Brien et al., 1993; Farr et al., 2002; Paula et al., 2005; Cornelius et al., 2013). Ouabain, in agreement with our dataset on insect Na^+^/K^+^-ATPase, had a higher inhibitory potency than ouabagenin on lamb-, shark-, and human Na^+^/K^+^-ATPase (Farr et al., 2002; Paula et al., 2005; Katz et al., 2010; Cornelius et al., 2013). As for adapted and non-adapted insect Na^+^/K^+^-ATPases, digoxin and digitoxin had a higher inhibitory potency on shark and human Na^+^/K^+^-ATPase (Cornelius et al., 2013) compared to digoxigenin and digitoxigenin, but the reverse was found for lamb Na^+^/K^+^-ATPase (Farr et al., 2002; Paula et al., 2005). Shark and lamb-Na^+^/K^+^-ATPase also showed opposing results for the comparison between gitoxin and gitoxigenin (Farr et al., 2002; Paula et al., 2005; Cornelius et al., 2013). Surprisingly, the glycoside cymarin did not cause differential inhibition from its corresponding genin strophanthidin on lamb-Na^+^/K^+^-ATPase (Farr et al., 2002), but was ninefold more inhibiting on *D. plexippus*-Na^+^/K^+^-ATPase (but see Paula et al., 2005). These comparisons indicate that the effect of the cardiac glycoside sugar moiety on inhibition depends on the specific biochemical properties of the receptor Na^+^/K^+^-ATPase. Additionally, by conducting the analyses of compound impacts on Na^+^/K^+^-ATPases in a single study, our results are more easily interpretable because of consistency in purity of compounds and methods employed. Across the five pairs of glycosides and genins, glycosides were on average sixfold more potent in their inhibition of insect Na^+^/K^+^-ATPases, including the specialized monarch.

The unusual features of sugars present in cardiac glycosides suggest an adaptive significance for plants, but the mechanisms of natural selection have not been revealed. Apart from absorption, these sugars could contribute to hydrolytic stability of cardiac glycosides which could be an anti-predator strategy (e.g., prevention of hydrolysis in the animal gut). In this regard, cardenolides from the Asclepiadoideae (the milkweeds), which have cyclic bridges, are highly resistant to acid hydrolysis (Seiber et al., 1983) and should thus receive specific future attention. Although some insects attach sugar moieties to toxic aglycones (genins) in the course of detoxification (Heckel, 2014), it is unclear whether this could be important for insects adapted to cardiac glycosides. Given the increase of Na^+^/K^+^-ATPase inhibition in glycosides vs. genins, we speculate that glycosylation of cardiac glycoside genins could lead to metabolic activation instead of detoxification.

Another remarkable feature of milkweed cardenolides is the *trans*-bent junction of rings A and B in the steroid (Seiber et al., 1983; Malcolm, 1991) which is *cis*-bent in *Digitalis* and other medicinally relevant cardenolides. Such *trans*-bent 5α-cardiac glycosides were speculated to have a highly reduced biological potency based on their toxicity (Brown and Thomas, 1984) and show low affinity to mammalian Na^+^/K^+^-ATPase (Paula et al., 2005; Katz et al., 2010). Although the direct comparison of uzarigenin with its 5β-isomer digitoxigenin revealed the A/B *trans*-ring junction as being causal for lower binding affinity and reduced inhibition on the mammalian enzyme (Farr et al., 2002; Paula et al., 2005), we found no differences in inhibitory strength between the two isomers, neither for non-adapted nor for adapted insect Na^+^/K^+^-ATPases. Moreover, the most toxic cardenolides observed, calactin and calotropin, also are 5α cardenolides. The putatively different effect of the A/B *trans*-ring junction on insect vs. vertebrate Na^+^/K^+^-ATPase should be addressed in future studies. Our observation that uzarigenin has a strongly reduced water solubility compared to digitoxigenin points to the importance of the configuration of the A/B ring junction for basic physicochemical properties and thus could easily influence absorption and sequestration of milkweed cardenolides in herbivorous insects.

How plants can respond selectively to attacks with defenses specific for particular antagonists is a major unanswered question in the study of plant–insect coevolution. It has been shown that structurally different plant defenses (e.g., monoterpenes from thyme, Linhart and Thompson, 1999) or condensed tannins from several plant families (Ayres et al., 1997) can affect different herbivores with different strength, but the underlying mechanisms have never been revealed. In his book on cardiac glycosides, Hoch (1961) states that “an arrangement of cardiac glycosides in the order of their relative toxicities would not be the same for different animal species.” Here, we show in a detailed mechanistic way, that structurally different cardiac glycosides can affect specific animal Na^+^/K^+^-ATPases in highly distinct ways. Our observation thus suggests a mechanistic basis for how such selectivity could be achieved on the level of plant toxins and their physiological targets. Moreover, the concept of coevolution predicts reciprocal escalation between plants and insects, and cardiac glycosides like uzarin which show comparatively little relative selectivity between the adapted *D. plexippus*-Na^+^/K^+^-ATPase and the non-adapted Na^+^/K^+^-ATPase, could be the plants’ response to the adapted insect’s target site (Farrell and Mitter, 1998). Indeed, there is evidence that isoforms of human Na^+^/K^+^-ATPase are also differentially inhibited by individual cardiac glycosides (Katz et al., 2010), which might be related to the importance of cardiac glycosides as endogenously occurring hormones and fine-scale regulation of tissue-specific isoforms of Na^+^/K^+^-ATPase. We speculate that the dramatically different effects which structurally diverse cardiac glycosides have on the more resistant insect Na^+^/K^+^-ATPases might be a driving force selecting for structural diversity of defense chemicals. For interactions involving sequestration, such as that between milkweeds and monarchs, both the plant and the animal antagonists must manage toxicity, not only in the pair but also to the third trophic level.

## Author Contributions

GP, CF, and SS collected data. AA, JA, and BT provided materials. GP and AA designed research, analyzed data, and wrote the paper.

## Conflict of Interest Statement

The authors declare that the research was conducted in the absence of any commercial or financial relationships that could be construed as a potential conflict of interest.

## References

[B1] Aardema M. L. Zhen Y. Andolfatto P. (2012). The evolution of cardenolide-resistant forms of Na^+^,K^+^-ATPase in Danainae butterflies. *Mol. Ecol.* 21 340–349. 10.1111/j.1365-294X.2011.05379.x 22126595

[B2] Agrawal A. A. (2000). Specificity of induced resistance in wild radish: causes and consequences for two specialist and two generalist caterpillars. *Oikos* 89 493–500. 10.1034/j.1600-0706.2000.890308.x

[B3] Agrawal A. A. (2005). Natural selection on common milkweed (*Asclepias syriaca*) by a community of specialized insect herbivores. *Evol. Ecol. Res.* 7 651–667.

[B4] Agrawal A. A. (2017). *Monarchs and Milkweed: A Migrating Butterfly, a Poisonous Plant, and Their Remarkable Story of Coevolution.* Princeton, NJ: Princeton University Press 10.1515/9781400884766

[B5] Agrawal A. A. Fishbein M. (2008). Phylogenetic escalation and decline of plant defense strategies. *Proc. Natl. Acad. Sci. U.S.A.* 105 10057–10060. 10.1073/pnas.0802368105 18645183 PMC2481309

[B6] Agrawal A. A. Petschenka G. Bingham R. A. Weber M. G. Rasmann S. (2012). Toxic cardenolides: chemical ecology and coevolution of specialized plant-herbivore interactions. *New Phytol.* 194 28–45. 10.1111/j.1469-8137.2011.04049.x 22292897

[B7] Ali J. G. Agrawal A. A. (2012). Specialist versus generalist insect herbivores and plant defense. *Trends Plant Sci.* 17 293–302. 10.1016/j.tplants.2012.02.006 22425020

[B8] Ali J. G. Agrawal A. A. (2014). Asymmetry of plant-mediated interactions between specialist aphids and caterpillars on two milkweeds. *Funct. Ecol.* 28 1404–1412. 10.1111/1365-2435.12271

[B9] Al-Robai A. A. Khoja S. M. Al-Fifi Z. I. (1990). Properties of ouabain-resistant Na^+^/K^+^-transporting ATPase from the excretory system of *Poekilocerus bufonius*. *Insect Biochem.* 20 701–707. 10.1016/0020-1790(90)90084-8 12213249

[B10] Araya J. J. Binns F. Kindscher K. Timmermann B. N. (2012a). Verticillosides A-M: polyoxygenated pregnane glycosides from *Asclepias verticillata* L. *Phytochemistry* 78 179–189. 10.1016/j.phytochem.2012.02.019 22445072

[B11] Araya J. J. Kindscher K. Timmermann B. N. (2012b). Cytotoxic cardiac glycosides and other compounds from *Asclepias syriaca*. *J. Nat. Prod.* 75 400–407. 10.1021/np2008076 22316168

[B12] Ayres M. P. Clausen T. P. MacLean S. F. Redman A. M. Reichardt P. B. (1997). Diversity of structure and antiherbivore activity in condensed tannins. *Ecology* 78 1696–1712. 10.2307/2266094

[B13] Berenbaum M. R. Nitao J. K. Zangerl A. R. (1991). Adaptive significance of furanocoumarin diversity in *Pastinaca sativa* (Apiaceae). *J. Chem. Ecol.* 17 207–215. 10.1007/bf00994434 24258446

[B14] Berenbaum M. R. Zangerl A. R. (1996). “Phytochemical Diversity,” in *Phytochemical Diversity and Redundancy in Ecological Interactions. Recent Advances in Phytochemistry*, eds Romeo J. T. Saunders J. A. Barbosa P. (Boston, MA: Springer), 1–24. 10.1007/978-1-4899-1754-6_1

[B15] Brower L. P. Van Zandt Brower J. Corvino J. M. (1967). Plant poisons in a terrestrial food chain. *Proc. Natl. Acad. Sci. U.S.A.* 57 893–898. 10.1073/pnas.57.4.893 5231352 PMC224631

[B16] Brown L. Thomas R. (1984). Comparison of the inotropic effects of some 5 alpha-cardenolides on guinea pig left atria. *Arzneimittelforschung* 34 572–574. 6540575

[B17] Castillo G. Cruz L. L. Tapia-López R. Olmedo-Vicente E. Carmona D. Anaya-Lang A. L. (2014). Selection mosaic exerted by specialist and generalist herbivores on chemical and physical defense of *Datura stramonium*. *PLoS One* 9:e102478. 10.1371/journal.pone.0102478 25051169 PMC4106780

[B18] Cheung H. T. A. Nelson C. J. Watson T. R. (1988). New glucoside conjugates and other cardenolide glycosides from the monarch butterfly reared on *Asclepias fruticosa* L. *J. Chem. Soc. Perkin Trans.* 1 1851–1857. 10.1039/P19880001851

[B19] Cornelius F. Kanai R. Toyoshima C. (2013). A structural view on the functional importance of the sugar moiety and steroid hydroxyls of cardiotonic steroids in binding to Na,K-ATPase. *J. Biol. Chem.* 288 6602–6616. 10.1074/jbc.M112.442137 23341448 PMC3585100

[B20] Dobler S. Dalla S. Wagschal V. Agrawal A. A. (2012). Community-wide convergent evolution in insect adaptation to toxic cardenolides by substitutions in the Na,K-ATPase. *Proc. Natl. Acad. Sci. U.S.A.* 109 13040–13045. 10.1073/pnas.1202111109 22826239 PMC3420205

[B21] Dobler S. Petschenka G. Wagschal V. Flacht L. (2015). Convergent adaptive evolution - how insects master the challenge of cardiac glycoside-containing host plants. *Entomol. Exp. Appl.* 157 30–39. 10.1111/eea.12340

[B22] Duffey S. S. (1977). “Arthropod allomones: chemical effronteries and antagonists,” in *Proceedings of the XV International Congress Entomology*, Washingt, DC 19–27.

[B23] Dyer L. A. Dodson C. D. Stireman J. O. Tobler M. A. Smilanich A. M. Fincher R. M. (2003). Synergistic effects of three piper amides on generalist and specialist herbivores. *J. Chem. Ecol.* 29 2499–2514. 10.1023/a:1026310001958 14682530

[B24] Erb M. Meldau S. Howe G. A. (2012). Role of phytohormones in insect-specific plant reactions. *Trends Plant Sci.* 17 250–259. 10.1016/j.tplants.2012.01.003 22305233 PMC3346861

[B25] Farr C. D. Burd C. Tabet M. R. Wang X. Welsh W. J. Ball W. J. (2002). Three-dimensional quantitative structure-activity relationship study of the inhibition of Na^+^,K^+^-ATPase by cardiotonic steroids using comparative molecular field analysis. *Biochemistry* 41 1137–1148. 10.1021/bi011511g 11802712

[B26] Farrell B. D. Mitter C. (1998). The timing of insect/plant diversification: might *Tetraopes* (Coleoptera: Cerambycidae) and *Asclepias* (Asclepiadaceae) have co-evolved? *Biol. J. Linn. Soc.* 63 553–577. 10.1111/j.1095-8312.1998.tb00329.x

[B27] Groeneveld H. W. Steijl H. Berg B. Elings J. C. (1990). Rapid, quantitative HPLC analysis of *Asclepias fruticosa* L. and *Danaus plexippus* L. cardenolides. *J. Chem. Ecol.* 16 3373–3382. 10.1007/BF00982104 24263435

[B28] Heckel D. G. (2014). “Insect detoxification and sequestration strategies,” in *Annual Plant Reviews*, eds Voelckel C. Jander G. (Hoboken, NJ: John Wiley & Sons, Ltd), 77–114. 10.1002/9781118829783.ch3

[B29] Hoch J. (1961). *A Survey of Cardiac Glycosides and Genins.* Charleston, SC: University of South Carolina Press.

[B30] Holzinger F. Frick C. Wink M. (1992). Molecular basis for the insensitivity of the monarch (*Danaus plexippus*) to cardiac glycosides. *FEBS Lett.* 314 477–480. 10.1016/0014-5793(92)81530-Y 1334851

[B31] Imai S. Murase H. Katori M. Okada M. Shigei T. (1965). A study on the structure-activity relationship of the cardiotonic steroids. *Jpn. J. Pharmacol.* 15 62–71. 10.1254/jjp.15.62 14346560

[B32] Johnson M. T. J. Stinchcombe J. R. (2007). An emerging synthesis between community ecology and evolutionary biology. *Trends Ecol. Evol.* 22 250–257. 10.1016/j.tree.2007.01.014 17296244

[B33] Jones C. G. Firn R. D. (1991). On the evolution of plant secondary chemical diversity. *Philos. Trans. Biol. Sci.* 333 273–280. 10.1098/rstb.1991.0077

[B34] Katz A. Lifshitz Y. Bab-Dinitz E. Kapri-Pardes E. Goldshleger R. Tal D. M. (2010). Selectivity of Digitalis glycosides for isoforms of human Na,K-ATPase. *J. Biol. Chem.* 285 19582–19592. 10.1074/jbc.M110.119248 20388710 PMC2885237

[B35] Kim J. H. Jander G. (2007). *Myzus persicae* (green peach aphid) feeding on *Arabidopsis* induces the formation of a deterrent indole glucosinolate. *Plant J.* 49 1008–1019. 10.1111/j.1365-313X.2006.03019.x 17257166

[B36] Kleine S. Müller C. (2011). Intraspecific plant chemical diversity and its relation to herbivory. *Oecologia* 166 175–186. 10.1007/s00442-010-1827-6 21053017

[B37] Komarnytsky S. Esposito D. Poulev A. Raskin I. (2013). Pregnane glycosides interfere with steroidogenic enzymes to down-regulate corticosteroid production in human adrenocortical H295R cells. *J. Cell. Physiol.* 228 1120–1126. 10.1002/jcp.24262 23065845 PMC3796370

[B38] Linhart Y. B. Thompson J. D. (1999). Thyme is of the essence: biochemical polymorphism and multi-species deterrence. *Evol. Ecol. Res.* 1 151–171.

[B39] Luckner M. Wichtl M. (2000). *Digitalis.* Stuttgart: Wissenschaftliche Verlagsgesellschaft mbH, 2000.

[B40] Maddox G. D. Root R. B. (1990). Structure of the encounter between goldenrod (*Solidago altissima*) and its diverse insect fauna. *Ecology* 71 2115–2124. 10.2307/1938625

[B41] Malcolm S. B. (1991). “Cardenolide-mediated interactions between plants and herbivores,” in *Herbivores: their Interactions with Secondary Plant Metabolites, the Chemical Participants*, 2nd Edn, Vol. I, eds Rosenthal G. A. Berenbaum M. R. (San Diego, CA: Academic Press), 251–296. 10.1016/B978-0-12-597183-6.50012-7

[B42] Mithen R. Raybould A. F. Giamoustaris A. (1995). Divergent selection for secondary metabolites between wild populations of *Brassica oleracea* and its implications for plant-herbivore interactions. *Heredity* 75 472–484. 10.1038/hdy.1995.164

[B43] Müller R. de Vos M. Sun J. Y. Sønderby I. E. Halkier B. A. Wittstock U. (2010). Differential effects of indole and aliphatic glucosinolates on lepidopteran herbivores. *J. Chem. Ecol.* 36 905–913. 10.1007/s10886-010-9825-z 20617455

[B44] O’Brien W. J. Wallick E. T. Lingrel J. B. (1993). Amino acid residues of the Na,K-ATPase involved in ouabain sensitivity do not bind the sugar moiety of cardiac glycosides. *J. Biol. Chem.* 268 7707–7712. 8385116

[B45] Parsons J. A. Summers R. J. (1971). Cat assay for the emetic action of digitalis and related glycosides (digitoxin, digoxin, lanatoside C, ouabain and calactin). *Br. J. Pharmacol.* 42 143–152. 10.1111/j.1476-5381.1971.tb07094.x 5580699 PMC1666988

[B46] Paula S. Tabet M. R. Ball W. J. (2005). Interactions between cardiac glycosides and sodium/potassium-ATPase: three-dimensional structure-activity relationship models for ligand binding to the E2-Pi form of the enzyme versus activity inhibition. *Biochemistry* 44 498–510. 10.1021/bi048680w 15641774

[B47] Petschenka G. Agrawal A. A. (2015). Milkweed butterfly resistance to plant toxins is linked to sequestration, not coping with a toxic diet. *Proc. R. Soc. B Biol. Sci.* 282:20151865. 10.1098/rspb.2015.1865 26538594 PMC4650158

[B48] Petschenka G. Fandrich S. Sander N. Wagschal V. Boppré M. Dobler S. (2013a). Stepwise evolution of resistance to toxic cardenolides via genetic substitutions in the Na^+^/K^+^-ATPase of milkweed butterflies (Lepidoptera: Danaini). *Evolution* 67 2753–2761. 10.1111/evo.12152 24033181

[B49] Petschenka G. Pick C. Wagschal V. Dobler S. (2013b). Functional evidence for physiological mechanisms to circumvent neurotoxicity of cardenolides in an adapted and a non-adapted hawk-moth species. *Proc. R. Soc. B Biol. Sci.* 280:20123089. 10.1098/rspb.2012.3089 23516239 PMC3619502

[B50] Petschenka G. Fei C. S. Araya J. J. Schröder S. Timmermann B. N. Agrawal A. A. (2018). *Data from: Relative Selectivity of Plant Cardenolides for Na^+^/K^+^-ATPases from the Monarch Butterfly and Non-Resistant Insects.* 10.5061/dryad.fq064 PMC6172315 30323822

[B51] Petschenka G. Offe J. K. Dobler S. (2012). Physiological screening for target site insensitivity and localization of Na^+^/K^+^-ATPase in cardenolide-adapted Lepidoptera. *J. Insect Physiol.* 58 607–612. 10.1016/j.jinsphys.2011.12.012 22343317

[B52] Petschenka G. Wagschal V. von Tschirnhaus M. Donath A. Dobler S. (2017). Convergently evolved toxic secondary metabolites in plants drive the parallel molecular evolution of insect resistance. *Am. Nat.* 190 S29–S43. 10.1086/691711 28731826

[B53] Poelman E. H. Kessler A. (2016). Keystone herbivores and the evolution of plant defenses. *Trends Plant Sci.* 21 477–485. 10.1016/j.tplants.2016.01.007 26832946

[B54] Rasmann S. Johnson M. D. Agrawal A. A. (2009). Induced responses to herbivory and jasmonate in three milkweed species. *J. Chem. Ecol.* 35 1326–1334. 10.1007/s10886-009-9719-0 20012168

[B55] Reichstein T. von Euw J. Parsons J. A. Rothschild M. (1968). Heart poisons in the monarch butterfly. *Science* 161 861–866. 10.1126/science.161.3844.861 4875496

[B56] Richards L. A. Dyer L. A. Forister M. L. Smilanich A. M. Dodson C. D. Leonard M. D. (2015). Phytochemical diversity drives plant-insect community diversity. *Proc. Natl. Acad. Sci. U.S.A.* 112 10973–10978. 10.1073/pnas.1504977112 26283384 PMC4568244

[B57] Roeske C. N. Seiber J. N. Brower L. P. Moffitt C. M. (1976). Milkweed cardenolides and their comparative processing by monarch butterflies (*Danaus plexippus* L.). *Recent Adv. Phytochem.* 10 93–167. 10.1007/978-1-4684-2646-5_3

[B58] Schönfeld W. Weiland J. Lindig C. Masnyk M. Kabat M. M. Kurek A. (1985). The lead structure in cardiac glycosides is 5 β,14 β-androstane-3 β,14-diol. *Naunyn Schmiedebergs Arch. Pharmacol.* 329 414–426. 10.1007/BF00496377 4033807

[B59] Seiber J. Lee S. Benson J. (1983). “Cardiac glycosides (cardenolides) in species of *Asclepias* (Asclepiadaceae),” in *Handbook of Natural Toxins, Plant and Fungal Toxins* Vol. I eds Keeler R. F. Tu A. T. (Amsterdam: Marcel Dekker), 43–83.

[B60] Seiber J. N. Tuskes P. M. Brower L. P. Nelson C. J. (1980). Pharmacodynamics of some individual milkweed cardenolides fed to larvae of the monarch butterfly (*Danaus plexippus* L.). *J. Chem. Ecol.* 6 321–339. 10.1007/BF01402911

[B61] Teuscher E. Lindequist U. (2010). *Biogene Gifte.* Stuttgart: Wissenschaftliche Verlagsgesellschaft Stuttgart.

[B62] Vaughan G. L. Jungreis A. M. (1977). Insensitivity of lepidopteran tissues to ouabain: physiological mechanisms for protection from cardiac glycosides. *J. Insect Physiol.* 23 585–589. 10.1016/0022-1910(77)90052-X

[B63] Yatime L. Laursen M. Morth J. P. Esmann M. Nissen P. Fedosova N. U. (2011). Structural insights into the high affinity binding of cardiotonic steroids to the Na^+^,K^+^-ATPase. *J. Struct. Biol.* 174 296–306. 10.1016/j.jsb.2010.12.004 21182963

[B64] Yoda A. (1973). Structure-activity relationships of cardiotonic steroids for the inhibition of sodium- and potassium-dependent adenosine triphosphatase. *Mol. Pharmacol.* 9 766–773. 4271632

[B65] Zhen Y. Aardema M. L. Medina E. M. Schumer M. Andolfatto P. (2012). Parallel molecular evolution in an herbivore community. *Science* 337 1634–1637. 10.1126/science.1226630 23019645 PMC3770729

[B66] Züst T. Heichinger C. Grossniklaus U. Harrington R. Kliebenstein D. J. Turnbull L. A. (2012). Natural enemies drive geographic variation in plant defenses. *Science* 338 116–119. 10.1126/science.1226397 23042895

[B67] Züst T. Rasmann S. Agrawal A. A. (2015). Growth-defense tradeoffs for two major anti-herbivore traits of the common milkweed *Asclepias syriaca*. *Oikos* 124 1404–1415. 10.1111/oik.02075

