# Supplementary material for: Relative Selectivity of Plant Cardenolides for Na+/K+-ATPases From the Monarch Butterfly and Non-resistant Insects
Source: Front Plant Sci. 2018 Sep 28;9:1424. doi: 10.3389/fpls.2018.01424 (PMC6172315; doi:10.3389/fpls.2018.01424)
Supplement: Supplementary file 1 [file Data_Sheet_1.doc]

**Relative Selectivity of Plant Cardenolides for Na^+^/K^+^-ATPases from the Monarch Butterfly and Non-Resistant Insects**

Georg Petschenka^1*^, Colleen S Fei^2^, Juan J Araya^3^, Susanne Schröder^4^, Barbara N Timmermann^5^, Anurag A Agrawal^2^

^1^Institute for Insect Biotechnology, Justus-Liebig-Universität, Giessen, Germany; ^2^Department of Ecology and Evolutionary Biology, Cornell University, Ithaca, NY, USA; ^3^Centro de Investigaciones en Productos Naturales, Escuela de Química; Instituto de Investigaciones Farmacéuticas, Facultad de Farmacia, Universidad de Costa Rica, San Pedro, Costa Rica; ^4^Institut für Medizinische Biochemie und Molekularbiologie, Universität Rostock, Rostock, Germany; ^5^Department of Medicinal Chemistry, School of Pharmacy, University of Kansas, Lawrence, KS, USA

***Corresponding author:** Georg Petschenka, Institute for Insect Biotechnology, Justus-Liebig-Universität, Giessen, Germany; phone: +49-641-99-37603;

e-mail: Georg.Petschenka@googlemail.com

**Keywords:** monarch butterfly, Na^+^/K^+^-ATPase, cardenolide, cardiac glycoside, phytochemical diversity, structure-activity relationship, toxin-receptor interaction, resistance

**
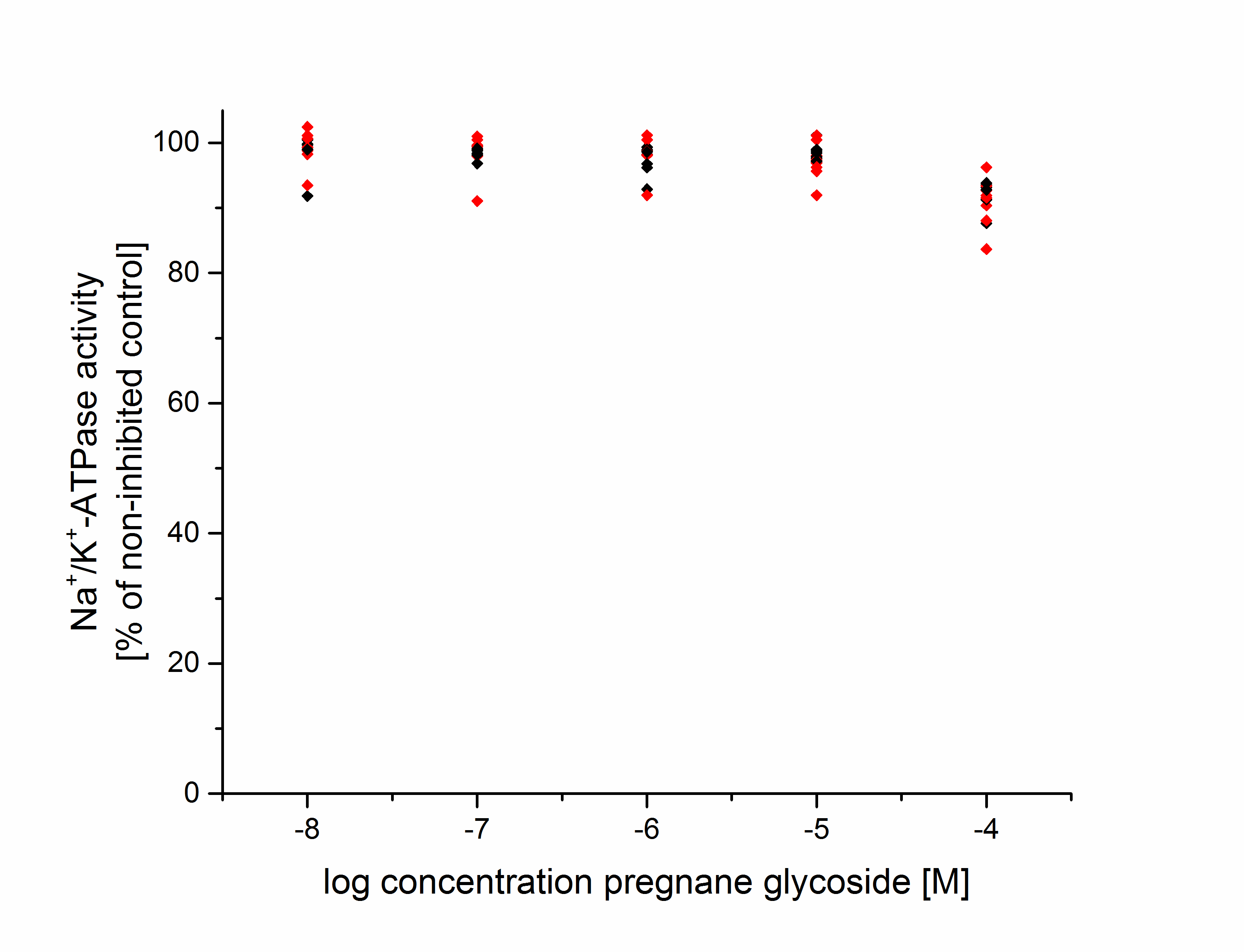
**

**Supplementary Figure 1:** Effect of the pregnane glycosides verticilloside A-H on *D. plexippus*- Na^+^/K^+^-ATPase (black) and *E. core* - Na^+^/K^+^-ATPase (red).

**
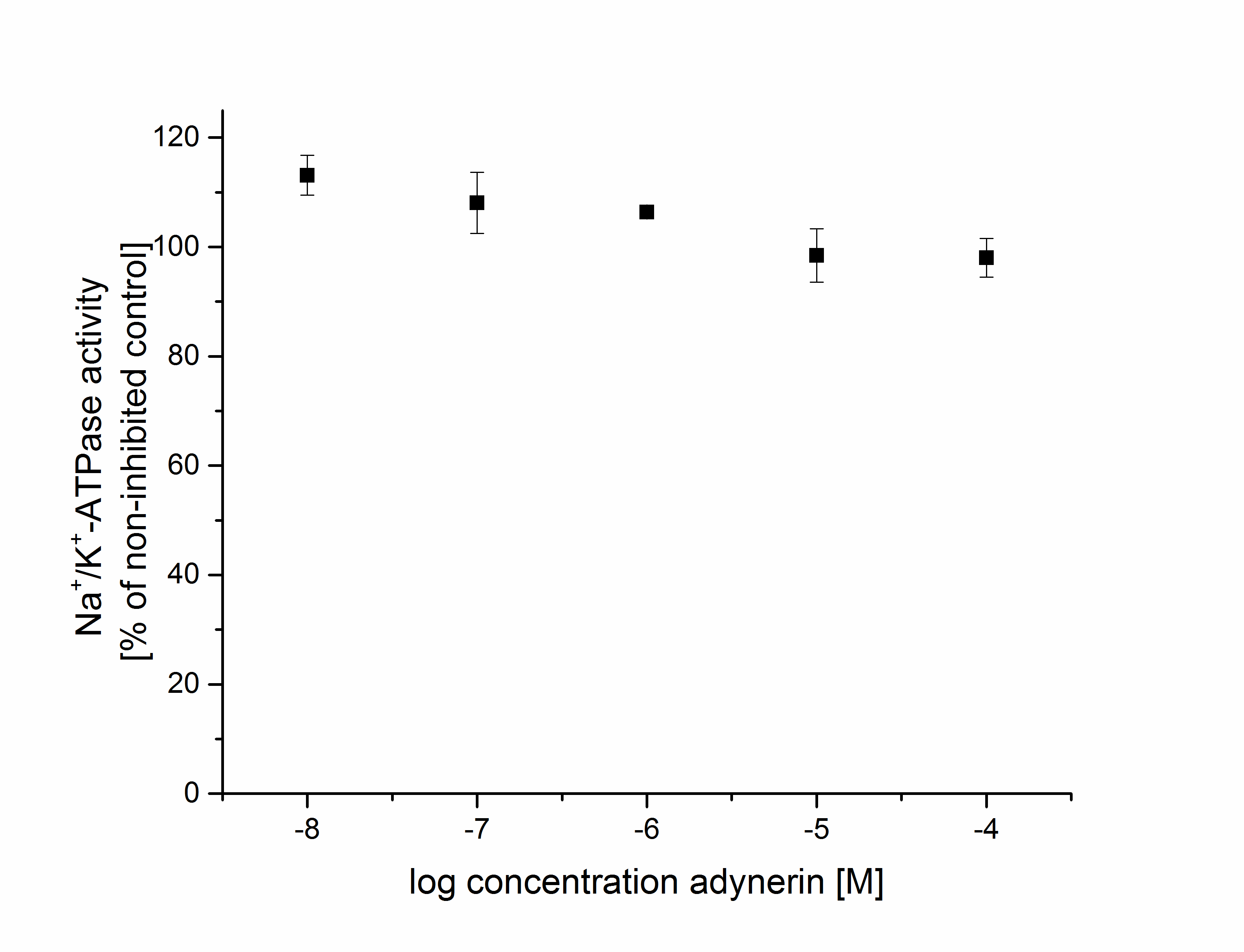
**

**Supplementary Figure 2**: Effect of adynerin on *S. gregaria*-Na^+^/K^+^-ATPase. Note the lack of inhibition.


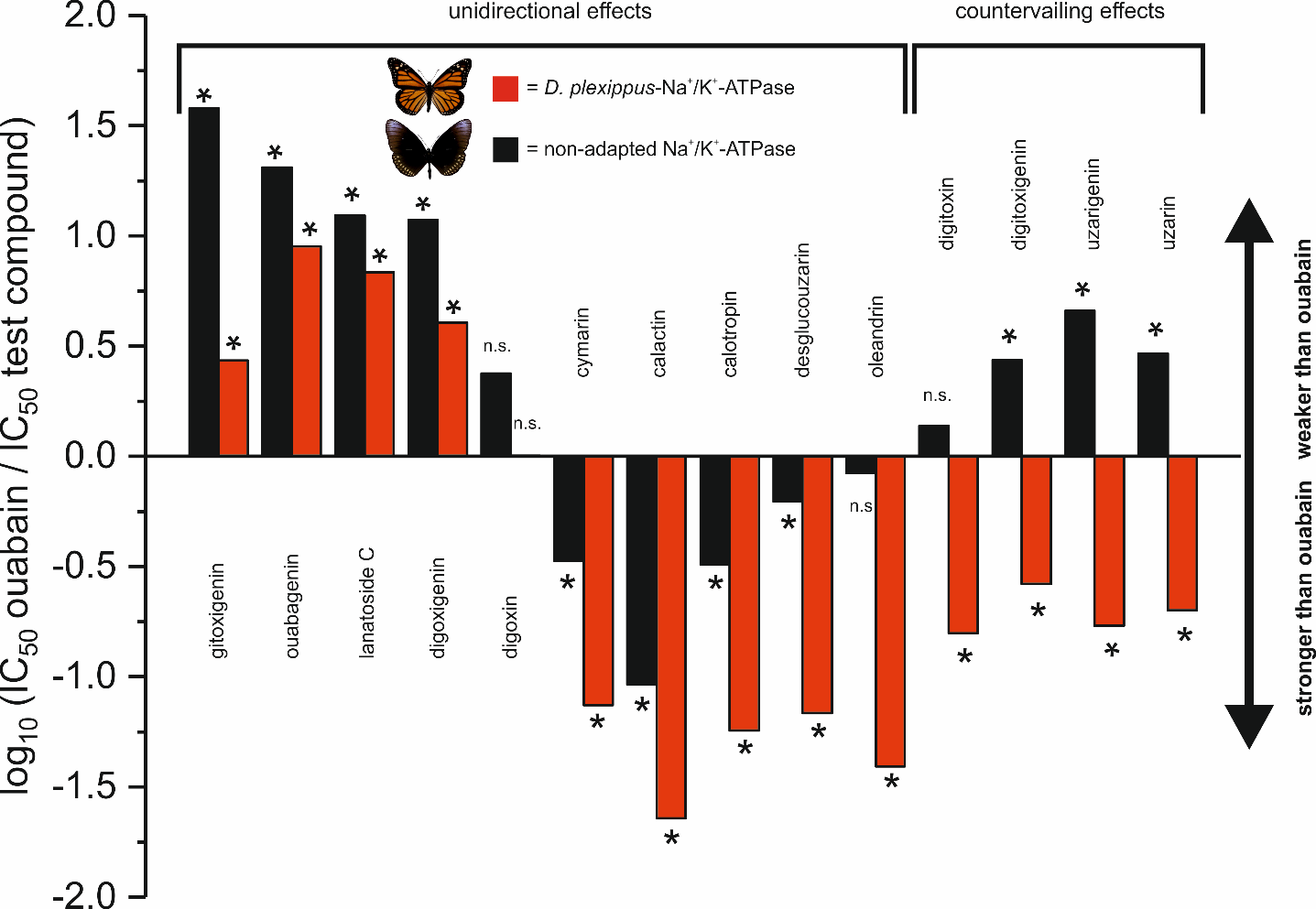


Supplementary Figure 3: Effects of cardenolides on adapted (*D. plexippus,* red) and non-adapted (*E. core* and *S. gregaria*, black) Na^+^/K^+^-ATPases. Inhibition based on IC_50_ values (Supplementary Table 1) is compared relative to the standard, ouabain. Log 10 transformation of the ratio of IC_50_ values (ouabain/test compound) results in inhibition weaker than ouabain indicated by negative values, while inhibition greater than ouabain is indicated by positive values. Specific interactions between Na^+^/K^+^-ATPases and cardenolides are most obvious in the five reversals (‘countervailing effects’ of digitoxin to uzarin), where cardenolides are more potent than ouabain on adapted Na^+^/K^+^-ATPase and less potent than ouabain on non-adapted Na^+^/K^+^-ATPase. Asterisks above columns indicate significant differences of the test compound compared to ouabain. Note that statistic comparisons are based on the comparison of entire curves and are not restricted to IC_50_ values only (see methods section and Supplementary Table 2).


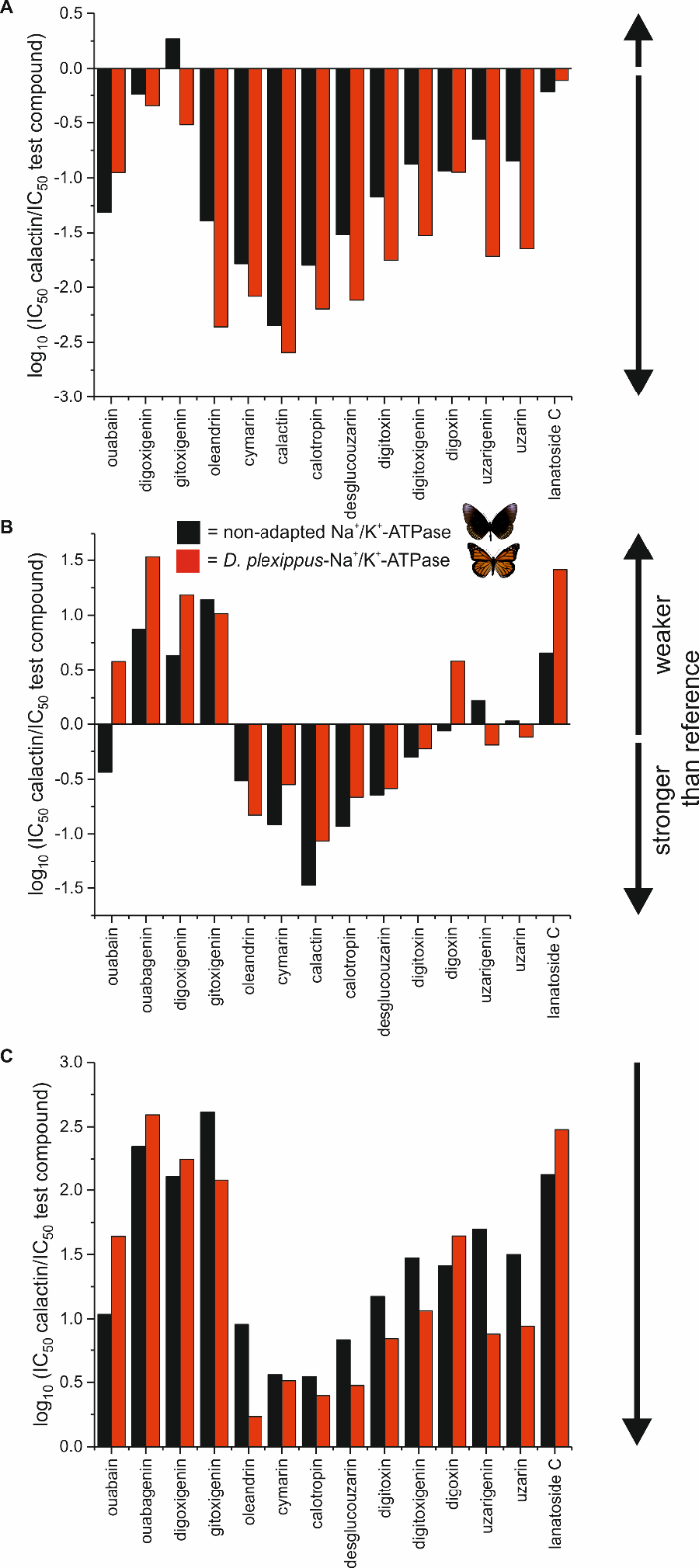


**Supplementary Figure 4**: Effects of individual cardenolides on adapted (*D. plexippus*, red) and non-adapted (*E. core* and *S. gregaria*, black) Na^+^/K^+^-ATPases. Inhibition based on IC_50_ values (Supplementary Table 1) is compared relative to three cardiac glycosides which encompass the range of inhibition observed. Log 10 transformation of the ratio of IC_50_ values (reference compound/test compound) results in inhibition weaker than the reference compound indicated by negative values, while inhibition greater than the reference compound is indicated by positive values. A: Ouabagenin (week toxicity) as reference compound. B: Digitoxigenin (intermediate toxicity) as reference compound. C: Calactin (high toxicity) as reference compound.


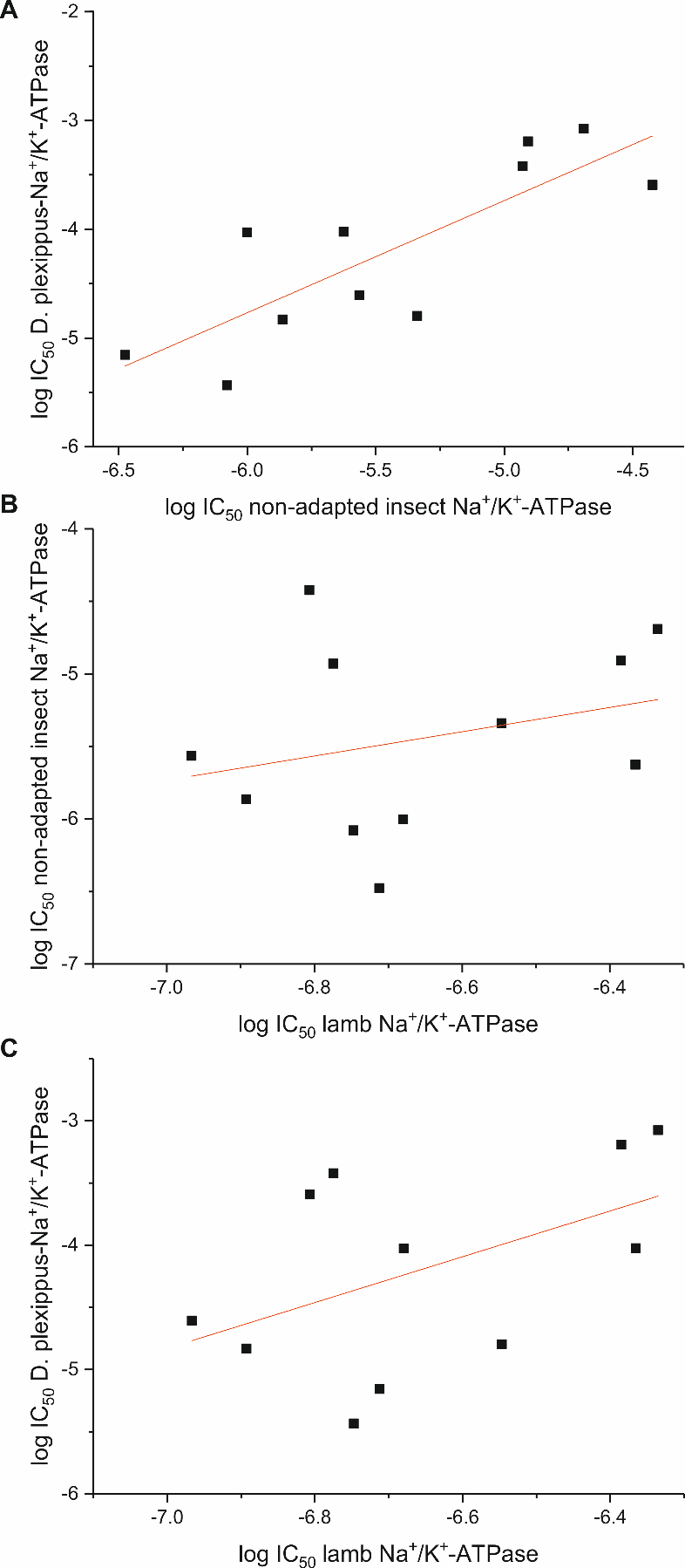


**Supplementary Figure 5**: Correlations between IC_50_ values of cardiac glycosides applied to two different forms of Na^+^/K^+^-ATPase. A: *D. plexippus*-Na^+^/K^+^-ATPase vs. *S. gregaria*/*E. core*- Na^+^/K^+^-ATPase. B: *S. gregaria*/*E. core*- Na^+^/K^+^-ATPase vs. lamb Na^+^/K^+^-ATPase (Paula et al., 2005). C: *D. plexippus*- Na^+^/K^+^-ATPase vs. lamb Na^+^/K^+^-ATPase.

**
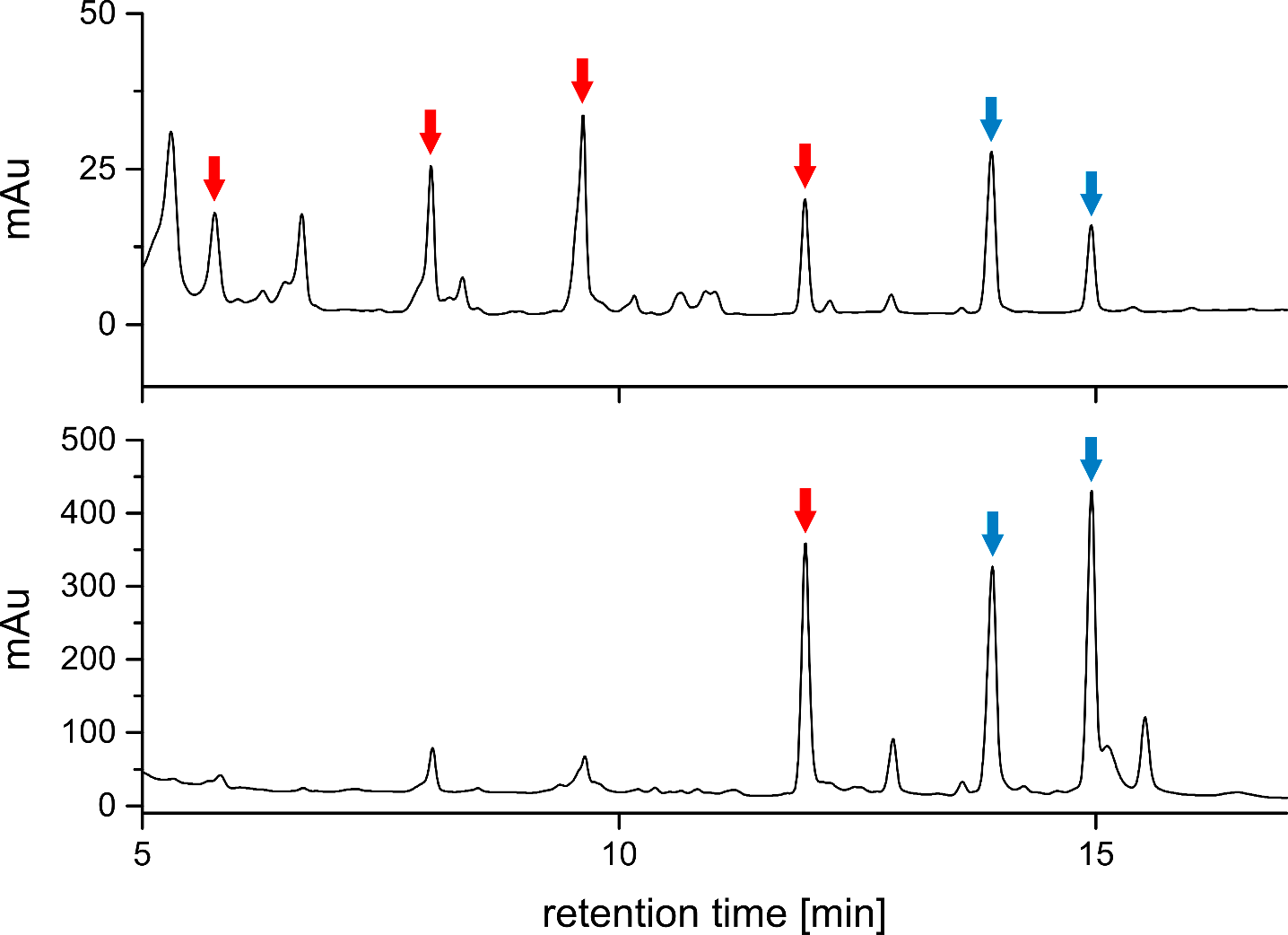
**

Supplementary Figure 6: HPLC-chromatograms showing cardenolide peaks in haemolymph derived from a monarch caterpillar raised on *Asclepias curassavica* (top panel) and from *D. plexippus*-wings (caterpillar also raised on *A. curassavica*, bottom panel). Arrows indicate the most abundant cardenolide peaks (absorption maximum at 220 nm). Blue arrows indicate calotropin (left) and calactin (right).

**
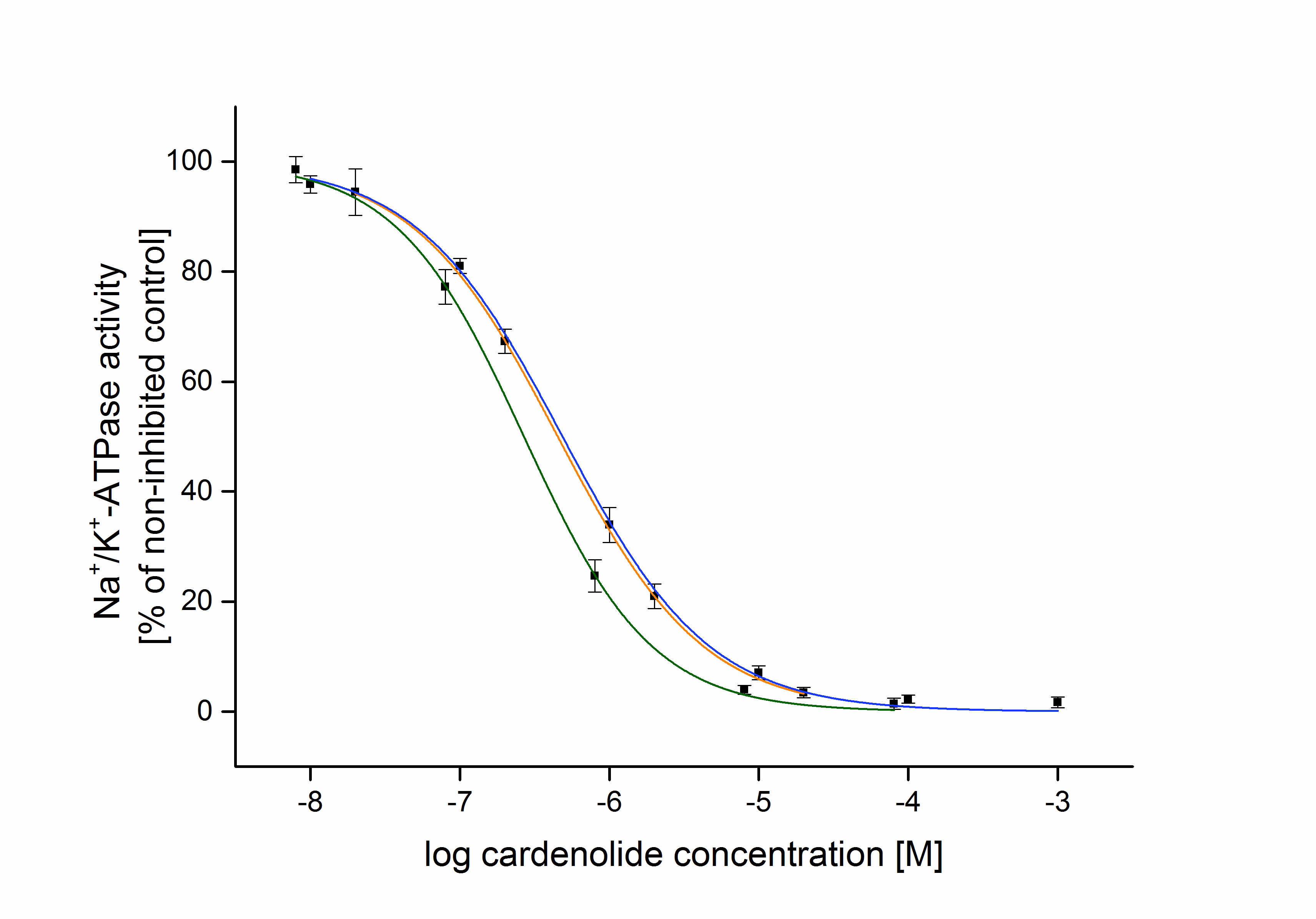
**

**Supplementary Figure 7**: *In vitro* inhibition of porcine Na^+^/K^+^-ATPase by ouabain (blue), uzarin (orange), and desglucouzarin (green). As the milkweed cardenolides uzarin and desglucouzarin produced strongly countervailing effects on adapted and non-adapted insect Na^+^/K^+^-ATPase compared to ouabain, we applied these cardenolides on a vertebrate (porcine) Na^+^/K^+^-ATPase for comparison. While porcine Na^+^/K^+^-ATPase was more sensitive to ouabain than the non-adapted Na^+^/K^+^-ATPase of *S. gregaria* or *E. core* (pig vs. *S. gregaria*: F_2_,_7_ = 24.05, p < 0.001), the effect of uzarin on porcine Na^+^/K^+^-ATPase was not different from the effect of ouabain (F_2_,_6_= 0.77, p = 0.5). Desglucouzarin, in contrast, inhibited porcine Na^+^/K^+^-ATPase more strongly than ouabain (F_2_,_7_ = 42.83, p < 0.001). While 5α-cardenolides such as uzarin were reported to have low affinity for human Na^+^/K^+^-ATPase (Katz et al., 2010) our data suggest that uzarin has a similar inhibitory potency like ouabain on porcine Na^+^/K^+^-ATPase and its monoside desglucouzarin showed even stronger inhibition.

**Supplementary Table 1**: IC_50_ values of 16 cardenolides and one bufadienolide (hellebrin) on insect and porcine Na^+^/K^+^-ATPase. Numbers in parentheses indicate the number of biological replicates (left) as well as the number of technical replicates (right). Na^+^/K^+^-ATPase of *E. core* was always investigated in parallel with *D. plexippus*-Na^+^/K^+^-ATPase on the same 96-well plate and we always ran an ouabain inhibition curve for both Na^+^/K^+^-ATPases as a standard alongside. For this reason, the level of technical replication for ouabain exceeds the number of replicates collected for any other cardiac glycoside. Although most of the IC_50_ values in this table are already presented in Figure 2, they are numeralized here for ease of interpretation.

| cardenolide | IC_50_ *S. gregaria*-  Na^+^/K^+^-ATPase [M] | IC_50_ *E. core*-  Na^+^/K^+^-ATPase [M] | IC_50_ *D. plexippus*-  Na^+^/K^+^-ATPase  [M] | IC_50_ porcine Na^+^/K^+^-ATPase [M] |
| --- | --- | --- | --- | --- |
| ouabain | 9.97 x 10^-7^ (3;6) | 1.03 x 10^-6^ (4;14) | 9.40 x 10^-5^ (7;17) | 4.86 x 10^-7^ (1;3) |
| ouabagenin | 2.04 x 10^-5^ (3;6) | - | 8.41 x 10^-4^ (3;6) |  |
| digitoxin | 1.37 x 10^-6^ (3;6) | - | 1.48 x 10^-5^ (3;6) |  |
| digitoxigenin | 2.73 x 10^-6^ (3;6) | 4.12 x 10^-6^ (3;3) | 2.48 x 10^-5^ (6;9) |  |
| digoxin | 2.37 x 10^-6^ (3;6) | - | 9.46 x 10^-5^ (3;6) |  |
| digoxigenin | 1.18 x 10^-5^ (3;6) | - | 3.79 x 10^-4^ (3;6) |  |
| cymarin | 3.34 x 10^-7^ (3;6) | - | 6.99 x 10^-6^ (3;6) |  |
| strophanthidin | - | - | 6.48 x 10^-5^ (3;6) |  |
| uzarin | - | 2.91 x 10^-6^ (3;3) | 1.88 x 10^-5^ (3) | 4.52 x 10^-7^ (1;3) |
| desglucouzarin | - | 6.20 x 10^-7^ (3;3) | 6.42 x 10^-6^ (3) | 2.68 x 10^-7^ (1;3) |
| uzarigenin | - | 4.58 x 10^-6^ (3;3) | 1.60 x 10^-5^ (3) |  |
| lanatoside c | 1.24 x 10^-5^ (3;6) | - | 6.44 x 10^-4^ (3;12) |  |
| gitoxigenin | 3.78 x 10^-5^ (3;6) | - | 2.56 x 10^-4^ (3;6) |  |
| oleandrin | 8.34 x 10^-7^ (3;6) | - | 3.68 x 10^-6^ (3;12) |  |
| calactin | - | 9.20 x 10^-8^ (3;3) | 2.15 x 10^-6^ (3) |  |
| calotropin | - | 3.23 x 10^-7^ (3;3) | 5.36 x 10^-6^ (3) |  |
| hellebrin | - | - | 8.42 x 10^-7^ (3;12) |  |
|  |  |  |  |  |

Supplementary Table 2: Statistic comparisons of inhibitors to ouabain

|  | DP | SG | EC |
| --- | --- | --- | --- |
| strophanthidin | > ouabain; F_2,6_ = 9.41,  p = 0.01 | n.d. | n.d. |
| oleandrin | > ouabain; F_2,6_ = 252.64,  p < 0.001 | = ouabain; F_2,6_ = 2.24,  p = 0.19 | n.d. |
| gitoxigenin | < ouabain; F_2,6_ = 8.49,  p = 0.02 | < ouabain; F_2,6_ = 109.1,  p < 0.001 | n.d. |
| digitoxin | > ouabain; F_2,6_ = 116.84,  p < 0.001 | = ouabain; F_2,6_ = 5.07,  p = 0.05 | n.d. |
| digitoxigenin | > ouabain; F_2,6_ = 54.05,  p < 0.001 | < ouabain; F_2,6_ = 17.5,  p = 0.003 | < ouabain; F_2,6_ = 180.27,  p < 0.001 |
| ouabagenin | < ouabain; F_2,6_ = 80.0,  p < 0.001 | < ouabain; F_2,6_ = 201.2,  p < 0.001 | n.d. |
| cymarin | > ouabain; F_2,6_ = 514.53,  p < 0.001 | > ouabain; F_2,6_ = 6.37,  p = 0.03 | n.d. |
| digoxin | > ouabain; F_2,6_ = 5.17,  p = 0.05 (0.0496) | = ouabain; F_2,6_ = 4.06,  p = 0.08 | n.d. |
| lanatoside C | < ouabain; F_2,6_ = 17.23,  p = 0.003 | < ouabain; F_2,6_ = 82.96,  p < 0.001 | n.d. |
| digoxigenin | < ouabain; F_2,6_ = 30.1,  p < 0.001 | < ouabain; F_2,6_ = 151.91,  p < 0.001 | n.d. |
| hellebrin | > ouabain; F_2,6_ = 1420.98,  p < 0.001 | n.d. | n.d. |
| calotropin | > ouabain; F_2,7_ = 154.4,  p < 0.001 | n.d. | > ouabain; F_2,7_ = 54.31,  p < 0.001 |
| calactin | > ouabain; F_2,7_ = 4706.35,  p < 0.001 | n.d. | > ouabain; F_2,7_ = 496.50,  p < 0.001 |
| uzarin | > ouabain; F_2,7_ = 70.04,  p < 0.001 | n.d. | < ouabain; F_2,7_ = 39.97,  p < 0.001 |
| desglucouzarin | > ouabain; F_2,7_ = 455.90,  p < 0.001 | n.d. | < ouabain; F_2,7_ = 19.34,  p = 0.001 |
| uzarigenin | > ouabain; F_2,6_ = 5.73,  p = 0.04 | n.d. | < ouabain; F_2,6_ = 53.30,  p < 0.001 |

Supplementary Table 3: Comparison of inhibition between cardenolide glycosides and their corresponding genins across adapted (*D. plexippus*)- and non-adapted Na^+^/K^+^-ATPase (*S. gregaria*/*E. core*).

|  | ouabain vs. ouabagenin | digoxin vs. digoxigenin | digitoxin vs. digitoxigenin | cymarin vs. strophanthidin | desglucouzarin vs. uzarigenin | uzarin vs. uzarigenin |
| --- | --- | --- | --- | --- | --- | --- |
| *D. plexippus* | F_2,6_ = 80.0  p < 0.001 | F_2,6_ = 70.36,  p < 0.001 | F_2,6_ = 7.86,  p = 0.02 | F_2,6_ = 121.41,  p < 0.001 | F_2,5_ = 158.43,  p < 0.001 | F_2,5_ = 1.12,  p = 0.4 |
| *S. gregaria* | F_2,6_ = 201.2,  p < 0.001 | F_2,6_ = 15.13,  p = 0.005 | F_2,6_ = 6.84,  p = 0.03 | - | - | - |
| *E. core* | - | - | - | - | F_2,5_ = 176.11,  p < 0.001 | F_2,5_ = 4.01,  p = 0.09 |
